# Hepatotoxic metabolites in *Polygoni Multiflori Radix*— Comparative toxicology in mice

**DOI:** 10.3389/fphar.2022.1007284

**Published:** 2022-10-11

**Authors:** Shixiao Wang, Xiang Kong, Ning Chen, Pengwei Hu, Hamza Boucetta, Zhaoliang Hu, Xin Xu, Pei Zhang, Xiang Zhan, Ming Chang, Rui Cheng, Wei Wu, Min Song, Yuting Lu, Taijun Hang

**Affiliations:** ^1^ Key Laboratory of Drug Quality Control and Pharmacovigilance (China Pharmaceutical University), Ministry of Education, Nanjing, China; ^2^ Department of Pharmaceutical Analysis, China Pharmaceutical University, Nanjing, China

**Keywords:** *Polygoni multiflori radix*, *rhei radix et rhizoma*, toxicology, hepatotoxicity, tissue distribution, correlation analysis

## Abstract

*Polygoni Multiflori Radix* (PM) and *Rhei radix et rhizoma* (rhubarb) contain similar hepatocyte-toxic anthraquinones such as emodin (major free anthraquinone in PM), physcion and their glycosides. In clinical practice, PM hepatotoxicity has been widely reported, although rhubarb is not recognized as hepatotoxic. To clarify the substances basis (key components) of PM hepatotoxicity, based on the characteristic components’ similarity within PM, rhubarb and their concocted forms, a comparative sub-acute toxicity study was designed in mice. Nine groups of mice with 28 days of oral administration of these herbal extracts or 2,3,5,4′-tetrahydroxystilbene-2-*O-β*-D-glucoside (TSG, major and unique characteristic component in PM)-herb combinations were set as follows: Group-1, control; Group-2, PM ethanol-extract (PME); Group-3, PM praeparata ethanol-extract (PMPE); Group-4, Rhubarb ethanol-extract (RME); Group-5, Steamed rhubarb ethanol-extract (RMPE); Group-6, TSG; Group-7, PMPE-TSG combination; Group-8, RME-TSG combination; Group-9, RMPE-TSG combination. Each experimental group received an equivalent emodin dose of 29 mg/kg except for the TSG group, and an equivalent TSG dose of 1,345 mg/kg except for the PMPE, RME and RMPE groups. The results showed that PME, PMPE-TSG and RME-TSG induced liver lesions and biochemical abnormalities of liver function compared with the control. In contrast, PMPE, RME, RMPE, TSG and RMPE-TSG caused no liver lesions and fewer biochemical abnormalities. Considering the related components, only the co-administration of high doses of TSG and emodin-8-*O-β*-D-glucoside (EMG, major anthraquinone glycoside in PM) in these groups could cause liver lesions. According to tissue distribution and correlation analysis, EMG dose was positively correlated with the high hepatic emodin and TSG exposure, and the hepatic emodin and TSG exposure were positively correlated with the biochemical abnormalities of liver function. Cell viability test *in vitro* showed emodin was more hepatotoxic than TSG and EMG, and mainly emodin and TSG of the three had synergistic hepatotoxic effects. Therefore, creatively using rhubarb as a reference, this study revealed that PM hepatotoxicity in mice mainly came from the integrative contribution of TSG, EMG and emodin.

## Introduction

*Polygoni Multiflori Radix* (PM) is the dried root of *Polygonum multiflorum* Thunb. (Polygonaceae family). According to the Chinese Pharmacopoeia, raw PM (3–6 g/person/day) is usually used for anti-infection, carbuncle elimination, and constipation relieving. As for *Polygoni Multiflori Radix Praeparata* (PMP) (6–12 g/person/day), prepared by steaming raw PM with black bean juice, it is used for treating hair graying, muscle and bone strengthening, hepatic/renal protection and blood lipid lowering. The main characteristic components in PM include TSG, free anthraquinones (emodin and physcion) and their corresponding glycosides (National Pharmacopoeia Committee, 2020) and these components are the main bioactive ingredients *in vivo* (Ma et al., 2021). In comparison with raw PM, its praeparata (PMP) has less TSG and anthraquinone glycosides, but has more emodin and physcion (Liu et al., 2009). Unfortunately, the occurrences of acute liver injury induced by PM have been documented in many countries worldwide (Mazzanti et al., 2004; Jung et al., 2011). Even the normal dose of PMP, which is generally deemed safe, can also cause liver damage (Li C. Y. et al., 2017). In recent years, it has increasingly attracted more attention in PM hepatotoxicity. However, it is still obscure and controversial in the hepatotoxic risk factors and components related to PM (Zhai et al., 2021). PM has a two-way effect of hepatoprotection and hepatotoxicity with uncertain critical dose (Li H. et al., 2017). As a result, large doses of PM are still used in clinical practice (Kong et al., 2022). Not only does it have idiosyncratic hepatotoxicity due to genetic polymorphism, but it also has inherent hepatotoxicity related to its main effective components such as the anthraquinones and stilbenes (Li et al., 2019; Liu et al., 2019). Because these related multiple components have big differences in contents *in vitro* and *in vivo*, the key hepatotoxic components (substances basis) are still necessary to be clarified.

*Rhei radix et rhizoma* (rhubarb), the dried root of *Rheum palmatum* L., *Rheum tanguticum* Maxim. Ex Balf. or *Rheum officinale* Baill. (Polygonaceae family), is also recorded in the Chinese Pharmacopoeia. Raw rhubarb (3–15 g/person/day) is used for heat clearing, jaundice treating, constipation relieving, blood stasis dissipating, and anti-infection. Its concocted form steamed rhubarb (3–15 g/person/day), prepared by steaming raw rhubarb with wine, is mainly used to clear away heat. Rhubarb is widely consumed in Europe as a laxative and it is also used in Asia as a weight-loss product. Anthraquinones are the main bioactive ingredients in rhubarb (Cao et al., 2017). Except for the main characteristic components including aloe-emodin, rhein and chrysophanol and their corresponding glycosides, rhubarb like PM also contains emodin, physcion and their corresponding glycosides. These emodin-type anthraquinones above have been recognized to have potential hepatotoxicity (He et al., 2019). Moreover, electrophilic intermediates produced by emodin, aloe-emodin and rhein in the anthraquinone components have been proven to damage the mitochondria and cause hepatocyte apoptosis *in vitro* (Bounda et al., 2015; Dong et al., 2017; Lin et al., 2019; Liu et al., 2020). However, no hepatotoxicity in humans has been reported for rhubarb, though its most prevalent side effects are diarrhea and constipation (Cao et al., 2017).

Emodin is the most hepatocyte-toxic component among the PM main anthraquinones (emodin, physcion and their main glycosides) (Lv et al., 2015), and it also possesses the potential to cause liver cholestasis, GSH depletion and nephrotoxicity (Yan et al., 2006; Jiang et al., 2017; Kang et al., 2017). TSG is the major and unique bioactive component in PM, and EMG is the main emodin glycoside and the major anthraquinone glycoside in PM (Yi et al., 2007; Wang et al., 2017). They are all related to the idiosyncratic hepatotoxicity of PM along with a synergistic effect (Zhang et al., 2020; Lin et al., 2021). In addition, on one hand, TSG could enhance the cell absorption of emodin by inhibiting its phase II metabolism and decreasing its efflux, on the other hand, TSG promoted emodin elimination by inducing its phase I metabolism (Qin et al., 2016; Xu et al., 2017; Yu et al., 2017). And EMG and other anthraquinone glycosides in rhubarb could be hydrolyzed to emodin and other corresponding free anthraquinones in the gastrointestinal tract, thereby increasing their exposure *in vivo* (Song et al., 2011). The potential toxicological effect and the pharmacokinetic interactions between multi-components in PM could be relevant to its herbal hepatotoxicity. Hence, the integral herbal toxicity differences between PM and rhubarb could stem from their component differences in kind and content.

**FIGURE 1:**
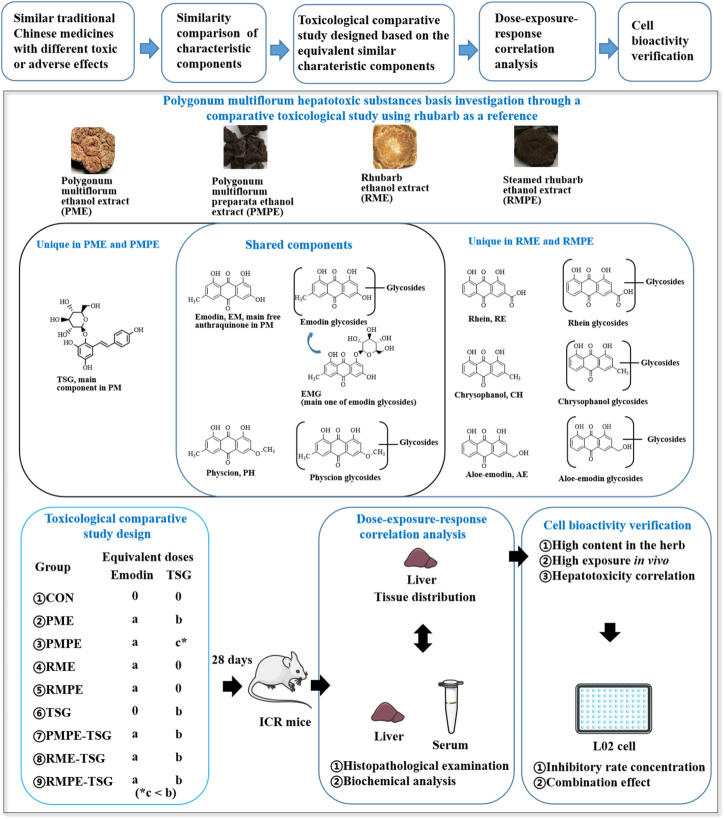
Scheme for the hepatotoxic substances basis study of PM using rhubarb as a reference based on their characteristic components’ similarity. ‘a, b, c’ represents the dose level of emodin or TSG. Each experimental group was designed to have an equivalent emodin dose of ‘a’ except for the TSG group, and an equivalent TSG dose of ‘b’ except for the PMPE (with TSG dose of ‘c’), RME and RMPE groups. In this PM hepatotoxicity study, ‘a’ was 29 mg/kg, ‘b’ was 1,345 mg/kg, ‘c’ was 346 mg/kg.

Therefore, in order to clarify the substances basis and mechanism of PM hepatotoxicity, a comparative sub-acute toxicity study, as shown in Figure 1, was designed based on the characteristic components’ similarity and different toxic potentials of PM, rhubarb and their concocted forms. Nine groups of mice with oral administration of these four herbal extracts and TSG-herb combinations for 28 days were set as follows: Group-1, CON; Group-2, PME; Group-3, PMPE; Group-4, RME; Group-5, RMPE; Group-6, TSG; Group-7, PMPE-TSG; Group-8, RME-TSG; Group-9, RMPE-TSG. Each experimental group was designed to have an equivalent emodin dose of 29 mg/kg except for the TSG group, and an equivalent TSG dose of 1,345 mg/kg except for the PMPE (with TSG dose of 346 mg/kg), RME and RMPE groups. Accordingly, the PME-, PMPE-, RME- and RMPE-containing groups had different equivalent EMG and other characteristic components doses. Toxicology differences were evaluated in these study groups through biochemical analysis and histopathological examination. Seven major characteristic components of PM or rhubarb in the mice tissues following the toxicological evaluation, including TSG, EMG, and five free anthraquinones (emodin, physcion, aloe-emodin, rhein, chrysophanol) were determined by a validated HPLC-MS/MS method. Then, Canonical or Pearson correlation analysis was performed to explore the dose-exposure-response correlation of these seven key components. In addition, the correlation between five kinds of anthraquinone glycosides (including the glycosides of emodin, physcion, aloe-emodin, rhein and chrysophanol) and the *in vivo* exposure of the corresponding free anthraquinones were also determined. A cell viability assay was conducted to assess the synergistic hepatotoxic effect of the three major characteristic components (emodin, TSG and EMG) in PM. The results are helpful for the rational clinical application of PM.

## Materials and methods

### Reagents and materials

*Polygoni Multiflori Radix* (Lot number: 20190301) was purchased from Sichuan Likun Chinese Medicine Co., Ltd. (Chengdu, China). *Polygoni Multiflori Radix Praeparata* (Lot number: 20190401) was purchased from Bozhou Yonggang Decoction Piece Factory (Bozhou, China). Both rhubarb and steamed rhubarb (*Rheum palmatum* L., Lot number: 20181101 and 20180301, respectively) were obtained from Anhui Jiuhetang Herbal Medicines Co., Ltd. (Bozhou, China). A representative specimen of each herb was deposited in the laboratory. TSG (content >90%) was obtained from Guilin Niutai Biotechnology Co., Ltd. (Guilin, China). TSG reference substance (content >98.0%) was obtained from Nanjing Daosifu Biotechnology Co., Ltd. (Nanjing, China). Reference substances of rhein, emodin, chrysophanol, aloe-emodin and physcion (all contents greater than 99.0%) were obtained from Chengdu Desite Biotechnology Co., Ltd. (Chengdu, China). 1,8-dihydroxyanthraquinone and EMG reference substances (content >99.0%) were obtained from Chengdu Pufeide Reference Technology Co., Ltd. (Chengdu, China). Commercial test kits for alanine aminotransferase (ALT), total bile acids (TBA), alkaline phosphatase (ALP), triglycerides (TG), total protein (TP), albumin (ALB), creatinine (CREA), glutathione (GSH), glutathione transferase (GST), malondialdehyde (MDA) were purchased from Nanjing Jiancheng Biotechnology Co., Ltd. (Nanjing, China). Mice tumor necrosis factor -α (TNF-α) enzyme-linked immunoassay kit was purchased from Elabscience Biotechnology (Wuhan, China). Both the BCA protein test kit and Cell Counting Kit-8 (CCK-8) kit were obtained from Shanghai Beyotime Biological Technology Co., Ltd. (Shanghai, China). Methanol and acetonitrile in chromatographical grade were purchased from TEDIA Co., Ltd. (Ohio, United States). Other reagents of analytical grade were purchased from Nanjing Chemical Reagent Co., Ltd. (Nanjing, China).

### Preparation of herbal extracts

**FIGURE 2:**
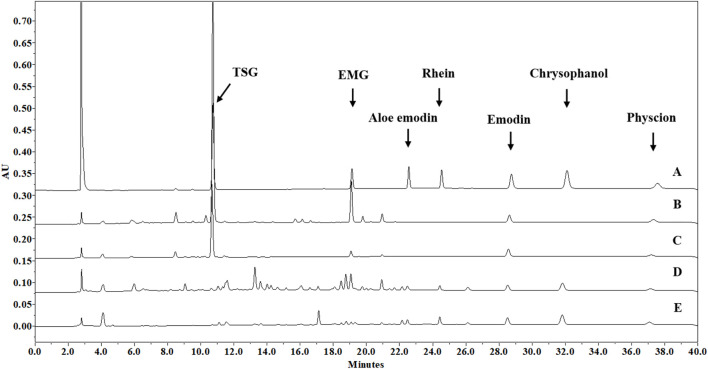
HPLC-UV chromatograms (275 nm) of seven major characteristic components in PM, rhubarb and their concocted herbal extracts. **(A)** Standard methanol solution, containing TSG (100 μg/ml), EMG (10 μg/ml), aloe-emodin (10 μg/ml), rhein (15 μg/ml), emodin (10 μg/ml), chrysophanol (10 μg/ml) and physcion (5 μg/ml). **(B)** PME methanol solution of 1.4 mg/ml (dry weight/methanol, w/v), containing TSG (228 μg/ml), EMG (17.3 μg/ml), emodin (4.86 μg/ml), and physcion (1.92 μg/ml); **(C)** PMPE methanol solution of 1.1 mg/ml (dry weight/methanol, w/v), containing TSG (57.6 μg/ml), EMG (2.91 μg/ml), emodin (4.80 μg/ml), and physcion (1.28 μg/ml); **(D)** RME methanol solution of 1.3 mg/ml (dry weight/methanol, w/v), containing EMG (6.24 μg/ml), emodin (3.61 μg/ml), physcion (1.30 μg/ml), aloe-emodin (1.34 μg/ml), rhein (2.83 μg/ml), and chrysophanol (6.82 μg/ml); **(E)** RMPE methanol solution of 0.8 mg/ml (dry weight/methanol, w/v), containing EMG (1.08 μg/ml), emodin (4.84 μg/ml), physcion (1.97 μg/ml), aloe-emodin (2.00 μg/ml), rhein (4.41 μg/ml) and chrysophanol (9.17 μg/ml).

Wine decoction was one of the traditional usage forms of PM, and ethanol was also used for PM extraction in many Chinese patent medicines. Therefore, an optimized ethanol extraction method was developed as follows: each herbal medicine was pulverized into small pieces, refluxed with 70% ethanol for 1 h at an herb-solvent ratio of 1:10 (w/v), and filtered while hot to collect the filtrate. The extraction was carried out for another two times using the same method. The collected filtrates were combined and evaporated by rotary evaporation under reduced pressure at 60°C to remove ethanol, the final concentrated solutions of PME, PMPE, RME and RMPE were determined to have a solid content of 0.43 ± 0.01, 0.41 ± 0.02, 0.66 ± 0.03 and 0.59 ± 0.04 g/ml through freeze-drying, respectively. Their major characteristic components, including TSG, EMG, and five free anthraquinones were determined by a validated HPLC method (Supplementary Table S1) for PM hepatotoxicity study. And the corresponding glycosides of five free anthraquinones were counted by the aglycones, which were equal to the total anthraquinones minus the free ones. The total anthraquinones were determined by the analytical method of free ones after the anthraquinone glycosides of the extract were hydrolyzed in 8% hydrochloric acid by referring to the Chinese Pharmacopoeia method. The contents of these key components in each herbal extract are shown in Table 1. HPLC-UV chromatograms (275 nm) of TSG, EMG and five free anthraquinones in PM, rhubarb and their concocted herbal extracts are shown in Figure 2.

**TABLE 1:** The contents of key components in PM, rhubarb and their concocted herbal extracts (μg/mg, mean ± SD, *n* = 2).

Extract	Extract rate (%)	TSG	EMG	EM	PH	AE	RE	CH	EM-G	PH-G	AE-G	RE-G	CH-G
PME	27.6 ± 0.8	162.4 ± 0.7	12.8 ± 0.7	3.49 ± 0.02	1.41 ± 0.04	—	—	—	10.7 ± 0.5	1.63 ± 0.11	—	—	—
PMPE	25.9 ± 1.0	52.9 ± 0.7	2.59 ± 0.08	4.44 ± 0.12	1.20 ± 0.04	—	—	—	1.89 ± 0.04	0.66 ± 0.04	—	—	—
RME	41.1 ± 2.0	—	4.82 ± 0.02	2.79 ± 0.01	1.01 ± 0.01	1.05 ± 0.02	2.00 ± 0.05	5.21 ± 0.05	4.43 ± 0.02	1.22 ± 0.07	2.12 ± 0.01	2.09 ± 0.02	5.77 ± 0.12
RMPE	39.3 ± 2.4	—	1.35 ± 0.01	6.01 ± 0.05	2.49 ± 0.05	2.54 ± 0.05	5.57 ± 0.08	11.53 ± 0.10	1.09 ± 0.03	0.59 ± 0.05	0.76 ± 0.03	0.89 ± 0.03	3.66 ± 0.08

EM, emodin; PH, physcion; AE, aloe-emodin; RE, rhein; CH, chrysophanol; EM-G, aglycones of hydrolyzed emodin glycosides; PH-G, aglycones of hydrolyzed physcion glycosides; AE-G, aglycones of hydrolyzed aloe-emodin glycosides; RE-G, aglycones of hydrolyzed rhein glycosides; CH-G, aglycones of hydrolyzed chrysophanol glycosides.

### Animal experiment design

Male ICR mice, 6 weeks old, 18–20 g in weight, were obtained from Shanghai SIPPR-BK Laboratory Animal Co., Ltd. (License number: SCXK 2018-0006) and raised under the controlled conditions (22°C–25°C, 30–70% relative humidity, and 12 h light/dark cycles) with free access to food and water in the Laboratory Animal Center of China Pharmaceutical University. All animal experiments were approved by the Animal Experiment Ethics Review Committee of China Pharmaceutical University (ethic approval number: 202105003). All operations followed the requirements of experimental animal welfare ethics of the Laboratory Animal Center of China Pharmaceutical University.

The comparative sub-acute toxicity investigation was carried out according to the ICH/M3 (R2) guideline (International Conference on Harmonization, 2009). Fifty-four healthy male ICR mice were randomly divided into 9 groups after acclimation for one week, with 6 mice in each group: 1) control (CON) group, 2) PME group, 3) PMPE group, 4) RME group, 5) RMPE group, 6) TSG group, 7) PMPE-TSG group, 8) RME-TSG group, 9) RMPE-TSG group. Suspensions of TSG, each herbal extract or each of the combinations were freshly prepared by diluting or dissolving them in normal saline with heating and stirring. Before use, the suspensions were stored in a water bath at 37°C to ensure their uniform dispersion. In this study, the dose of PME for mice was equivalent to 38 times the clinical maximum dose of 6 g/day raw PM for a 70 kg adult recommended by the current Chinese Pharmacopoeia (the dose conversion factor of body surface area from humans to mice is 9.1) according to the literature to uncover any potential toxicity (Ma et al., 2015; Ruan et al., 2019). Based on PME dose, the comparative study was designed by setting the equivalent emodin dose of each extract or the equivalent TSG dose of the TSG-herbal extract combinations in parallel (Table 2). During this experiment, the alterations in body weight of the mice in each group were recorded. Mice were sacrificed 1 h after dosing on the last day of the experiment, and then the blood and tissue samples were collected for the toxicological evaluation and tissue distribution study.

**TABLE 2:** Experimental groups and dosing regimen for PM hepatotoxicity study in ICR mice.

Group	Extract dose (g/kg)	TSG dose (g/kg)	Clinical dose for a 70 kg adult (raw herbs g/day)	Equivalent maximum clinical dose (times)	Equivalent key components dose (mg/kg)											
					TSG	EMG	EM	PH	AE	RE	CH	EM-G	PH-G	AE-G	RE-G	CH-G
CON	—	—	—	—	—	—	—	—	—	—	—	—	—	—	—	—
PME	8.3	—	3–6	38	1,345	106	29	12	—	—	—	89	13	—	—	—
PMPE	6.5	—	6–12	16	346	17	29	8	—	—	—	12	4	—	—	—
RME	10.4	—	3–15	13	—	50	29	10	11	21	54	46	13	22	22	60
RMPE	4.8	—	3–15	6	—	7	29	12	12	27	56	5	3	4	4	18
0.9%TSG	—	1.494	—	—	1,345	—	—	—	—	—	—	—	—	—	—	—
PMPE-TSG	6.5	0.999	—	—	1,345	17	29	8	—	—	—	12	4	—	—	0
RME-TSG	10.4	1.494	—	—	1,345	50	29	10	11	21	54	46	13	22	22	60
RMPE-TSG	4.8	1.494	—	—	1,345	7	29	12	12	27	56	5	3	4	4	18

The extracts, TSG, or TSG-herb combinations prepared in suspensions were administered by oral gavage of 19 ml/kg (about 0.6 ml) to the mice once per day for 28 consecutive days. An equal volume of normal saline was administered to the control mice in parallel. EM, emodin; PH, physcion, AE: aloe-emodin; RE, rhein, CH, chrysophanol; EM-G, aglycones of hydrolyzed emodin glycosides; PH-G, aglycones of hydrolyzed physcion glycosides; AE-G, aglycones of hydrolyzed aloe-emodin glycosides; RE-G, aglycones of hydrolyzed rhein glycosides; CH-G, aglycones of hydrolyzed chrysophanol glycosides.

### Histopathological examination and biochemical analysis

The necropsies were performed on all mice after oral administration for 28 days in this PM hepatotoxicity study, and the liver or kidney specimens from the same site in each mouse were immediately fixed in paraformaldehyde fixative, embedded in paraffin and sliced to a thickness of 5 µM. After staining with hematoxylin-eosin (HE), each tissue section was finally observed and photographed under a light microscope at 200 × magnification.

Liver homogenates at 10% were prepared with ice-cold normal saline, and intrahepatic GSH, GST, MDA were analyzed according to the requirements of test kits. Hepatic TNF-α levels were determined by the mice TNF-α enzyme-linked immunoassay kit. The BCA protein test kit was used for the calibration of biochemical indexes.

The mice serum samples were separated from the blood stood at room temperature over four hours with centrifugation at 1,100 × *g* for 10 min, and then the levels of serum ALT, TBA, ALP, TG, TP, ALB and CREA were determined by the commercial test kits. Serum globulin (GLB) level was equal to TP level minus ALB level.

### HPLC-MS/MS analytical method development

HPLC-MS/MS analysis for the seven key components (TSG, EMG and five free anthraquinones) in mice tissues of this PM hepatotoxicity study was conducted with a Thermo Dionex Ultimate 3000 HPLC system hyphenated with a TSQ Quantum Ultra AM triple quadrupoles mass spectrometer. The chromatographic separation was carried out on an Absolution C18 column (4.6 × 150 mm, 3 μm) with acetonitrile-water-acetic acid mixtures at ratios of 90:10:0.1 and 10:90:0.1 as mobile phases A and B, respectively, in linear gradient elution mode (A:B): 0 min (0:100) → 5 min (100:0) → 10 min (100:0) → 10.5 min (0:100) → 12 min (0:100), using the flow rate of 1.0 ml/min and column temperature of 35°C. The sample tray temperature was set at 15°C to obtain better solubility and stability. The injection volume was 20 μL. The MS/MS determination was performed using electrospray-negative ionization in multiple reaction monitoring (MRM) mode using an LC effluent split introduction ratio of 7:3. The optimized mass spectrometry parameters were as follows: spray voltage at −3,500 V, vaporizer temperature at 250°C, capillary temperature at 350°C, sheath gas (N2) pressure 344.7 kPa, collision gas (Ar) pressure 0.16 Pa, sweep gas (N2) pressure 3.4 kPa, and auxiliary gas (N2) pressure 34.5 kPa. The MRM transitions of TSG, EMG, emodin, aloe-emodin, rhein, chrysophanol, physcion and 1,8-dihydroxyanthraquinone (internal standard, IS) were *m*/*z* 405.00 @22 eV → 242.89, m/*z* 431.07 @29 eV → 268.88, m/*z* 269.00 @26 eV → 224.88, m/*z* 269.10 @23 eV → 239.87, m/*z* 283.10 @30 eV → 182.90, m/*z* 253.00 @27 eV →224.80, m/*z* 283.20 @25 eV →240.34 and *m*/*z* 239.00 @28 eV →210.97, respectively. Their chemical structures and MS/MS spectra are shown in Supplementary Figure S1.

The method validation for this HPLC-MS/MS analysis was performed according to the ICH bioanalysis guideline (International Conference on Harmonization, 2022), including specificity, linearity and sensitivity, accuracy and precision, matrix effect and recovery, and stability under different conditions (three freeze-thaw cycles, at room temperature for 8 h, and in the autosampler at 15°C for 24 h and storing at −80°C for 20 days). The method validation results are summarized in Supplementary Figures S2, S3 and Supplementary Tables S2–S4.

### Tissue distribution study

Following the toxicological evaluation in this PM hepatotoxicity study, the remaining mice tissue samples (liver, kidney, heart, spleen, lung, brain, stomach, large intestine and small intestine) were accurately weighed and homogenized in ice-cold normal saline at a weight-volume ratio of 1:4 to prepare the tissue homogenates. Then, an aliquot of 50 μL tissue homogenate sample was spiked with 10 μL of 1,000 ng/ml internal standard and 10 μL of 20% ascorbic acid in a 1.5 ml centrifuge tube, vortex-mixed with 0.4 ml methanol for 3 min, followed by centrifugation at 16,000 ×*g* for 10 min at 15°C. The supernatant was collected and evaporated to dryness in a vacuum centrifugation concentrator at 37°C. The residue was fully reconstituted with 0.12 ml of 80% methanol solution by vortex for 3 min and 20 μL of the resulting supernatant after another centrifugation was injected for the analysis of the seven key components in the tissues.

Appropriate amounts of TSG, EMG, emodin, physcion, aloe-emodin, rhein and chrysophanol or IS reference compounds were accurately weighed and dissolved using DMSO separately, and then diluted with methanol to prepare stocking solutions. The stocking solutions were then diluted into a series of mixed reference or IS working solutions. Following that, an aliquot of 50 μL blank liver homogenate was accurately mixed with 10 μL of a series of mixed reference solutions, and operated in parallel with sample preparation to prepare the calibration standards and quality control (QC) samples. The final concentration ranges of calibration standard samples were 0.4–4,000 ng/ml for TSG, 0.3–3,000 ng/ml for rhein, 0.1–800 ng/ml for emodin and EMG, 0.2–200 ng/ml for chrysophanol, aloe-emodin, and physcion. Four from low to high concentrations of QC samples prepared in the same way were as follows: 1, 10, 1,000 and 3,200 ng/ml for TSG, 0.75, 7.5, 600 and 2,400 ng/ml for rhein, 0.25, 2.5, 200 and 640 ng/ml for emodin and EMG, 0.5, 5, 80 and 160 ng/ml for chrysophanol, aloe-emodin, and physcion, respectively.

### Evaluation of hepatotoxicity of three key components and their combinations *in Vitro*

The normal human liver cell line L02 (purchased from the Cell Bank of Chinese Academy of Sciences, Shanghai, China) was cultured in DMEM medium with 1% penicillin–streptomycin (Keygen Biotech, China) supplemented with 10% fetal bovine serum (FBS, Biological Industries, Israel) at 37°C in a humidified incubator with 5% CO_2_.

The inhibitory effects of the three key compounds (TSG, EMG and emodin) and their different combinations on L02 cells were determined using a CCK-8 assay. The orthogonal experimental design is a fractional factorial design conducted by a Taguchi orthogonal array, which uses an orthogonal table to organize multiple factors and their different levels (Zhang et al., 2017). An orthogonal table L_9_ (3^4^) was used in this combined cell inhibitory test, corresponding to a total of 9 experiments involving four factors (TSG, EMG, emodin and random factor) and three levels (high, medium and low concentration levels). Briefly, the cells were cultured for 24 h on a 96-well plate (2.5×10^3^ cells/well), after which they were treated with TSG, EMG or emodin at different concentrations, or the nine orthogonal combinations of these three components at high, medium and low concentrations for 48 h. The medium was then removed and 10% CCK-8 working solution was added to each well, followed by incubation for 1 h. The optical density (OD) of each well was measured at 450 nm using a microplate reader (Thermo Fisher, United States). Cell viability was calculated as follows: cell viability (%) = (OD sample–OD blank)/(OD control–OD blank) × 100.

### Data analysis

All results were expressed as mean ± SD. SPSS23 was used for two-tailed independent Student’s *t*-test between two groups, Pearson correlation analysis between two variables, canonical correlation analysis (CCA) between two sets of variables and one-way ANOVA of the general linear model in orthogonal experiment. When *p* < 0.05, it was considered that there was a statistical significance. Graphpad prism was used to draw various graphs, and to fit each concentration-cell viability curve to get the half-inhibitory rate concentration (IC50) value. Three compounds’ combination inhibitory effect on L02 cells *in vitro* was assessed by Combination Index (CI) calculated by CompuSyn. A mild synergistic effect was considered when CI < 0.9, a mild antagonistic effect was considered when CI > 1.1, and an additive effect was considered when 0.9 < CI < 1.1 (Ashton, 2015).

## Results

### Liver/kidney toxicity in mice of PM ethanol extract and each comparison material

#### Body weight and histopathological alterations

**FIGURE 3:**
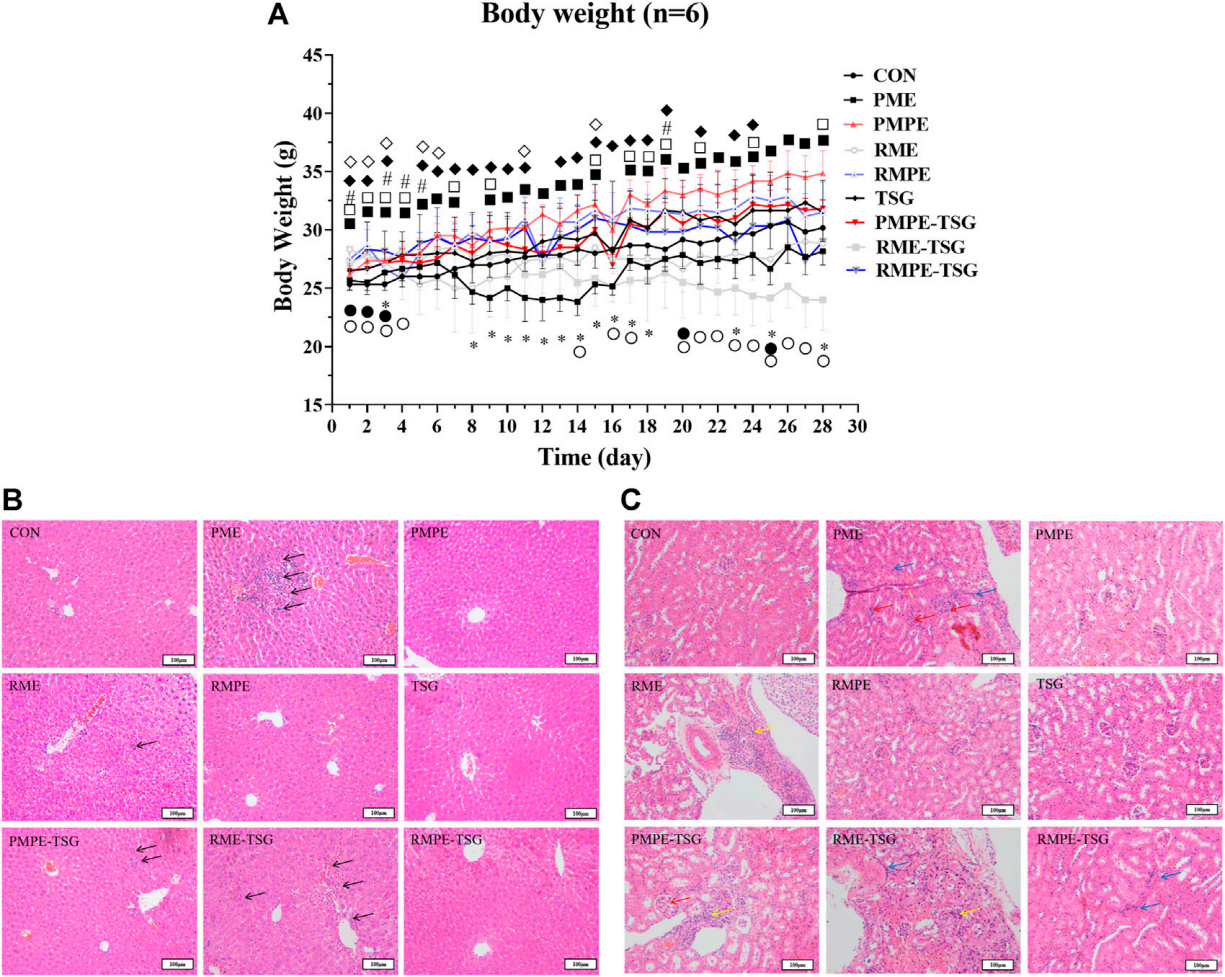
Alterations in body weight and liver and kidney morphology in mice of each group in PM hepatotoxicity study. **(A)** The mean growth curves of mice in all study groups. Data were expressed as mean ± SD (*n* = 6); Two-tailed independent Student’s *t*-test was conducted between each experimental group and the CON group. When *p* < 0.05, there was a statistical significance. *: *p* < 0.05, PME group; ■: *p* < 0.05, PMPE group; ●: *p* < 0.05, RME group; ♦: *p* < 0.05, RMPE group; #: *p* < 0.05, TSG; □: *p* < 0.05, PMPE-TSG group; ○: *p* < 0.05, RME-TSG group; ◊: *p* < 0.05, RMPE-TSG group. **(B)** Typical liver histological section photos of mice in each study group. The black arrow ↑ represents inflammatory cell infiltration or cell necrosis. **(C)** Typical kidney histological section photos of mice in each study group. The green arrow ↑ represents renal tubular epithelial cell necrosis and renal interstitial inflammatory cell infiltration, the red arrow ↑ represents the increase of glomerular cells and the atrophy of the glomerular capsule, and the blue arrow ↑ represents the increase of renal interstitial fibroblasts. Group-1: CON, Group-2: PME (8.3 g/kg), Group-3: PMPE (6.5 g/kg), Group-4: RME (10.4 g/kg), Group-5: RMPE (4.8 g/kg), Group-6: TSG (1,345 mg/kg), Group-7: PMPE-TSG, Group-8: RME-TSG, Group-9: RMPE-TSG. Each experimental group was designed to have an equivalent emodin dose of 29 mg/kg except for the TSG group, and an equivalent TSG dose of 1,345 mg/kg except for the PMPE (with TSG dose of 346 mg/kg), RME and RMPE groups. Thereby, the PME-, PMPE-, RME- and RMPE-containing groups had different EMG doses (106, 17, 50 and 7 mg/kg, respectively) and other characteristic components doses. The details about the dosage regime of this PM hepatotoxicity study are shown in the “Animal experiment design” part.

The mice growth curves of all the groups after 28 days of oral administration of PME and each comparison material are shown in Figure 3A. The mean growth rates of mice in PME, RME and RME-TSG groups were slower than that in CON group. After the initial three days of gavage, these three experimental groups developed obvious diarrhea. This could be caused by the laxative effect of anthraquinone glycosides (Qin et al., 2011). In contrast, the remaining groups grew faster than the CON group during most of the experiment. In addition, the combination groups had slower mean growth rates than the corresponding herbal extract-only groups, especially during the late experimental phase, indicating that the combinations might lead to stronger toxicity.

Histopathological examination represents a gold standard for disease diagnosis. As depicted in Figure 3B, the PMPE, TSG, RMPE and RMPE-TSG groups did not display any significant changes in liver lobular architecture and cell structure compared with the CON group. In contrast, the PME, PMPE-TSG and RME-TSG groups showed slight inflammation and multiple punctiform necrosis in the portal area, these were in consistence with pathological features of liver lesions. Mice in the RME group also displayed occasional punctiform necrosis.

PM may cause kidney toxicity by affecting the amino acid metabolism, which in turn accelerates liver damage (Gong et al., 2019), thus the kidney toxicity study was performed at the same time. Figure 3C displays typical kidney histological features in different groups. There were no notable differences between the PMPE, TSG or RMPE groups and the CON group. In contrary, mild renal pathological morphology alterations were all observed in the remaining groups.

#### Serum and hepatic biochemical abnormalities

**FIGURE 4:**
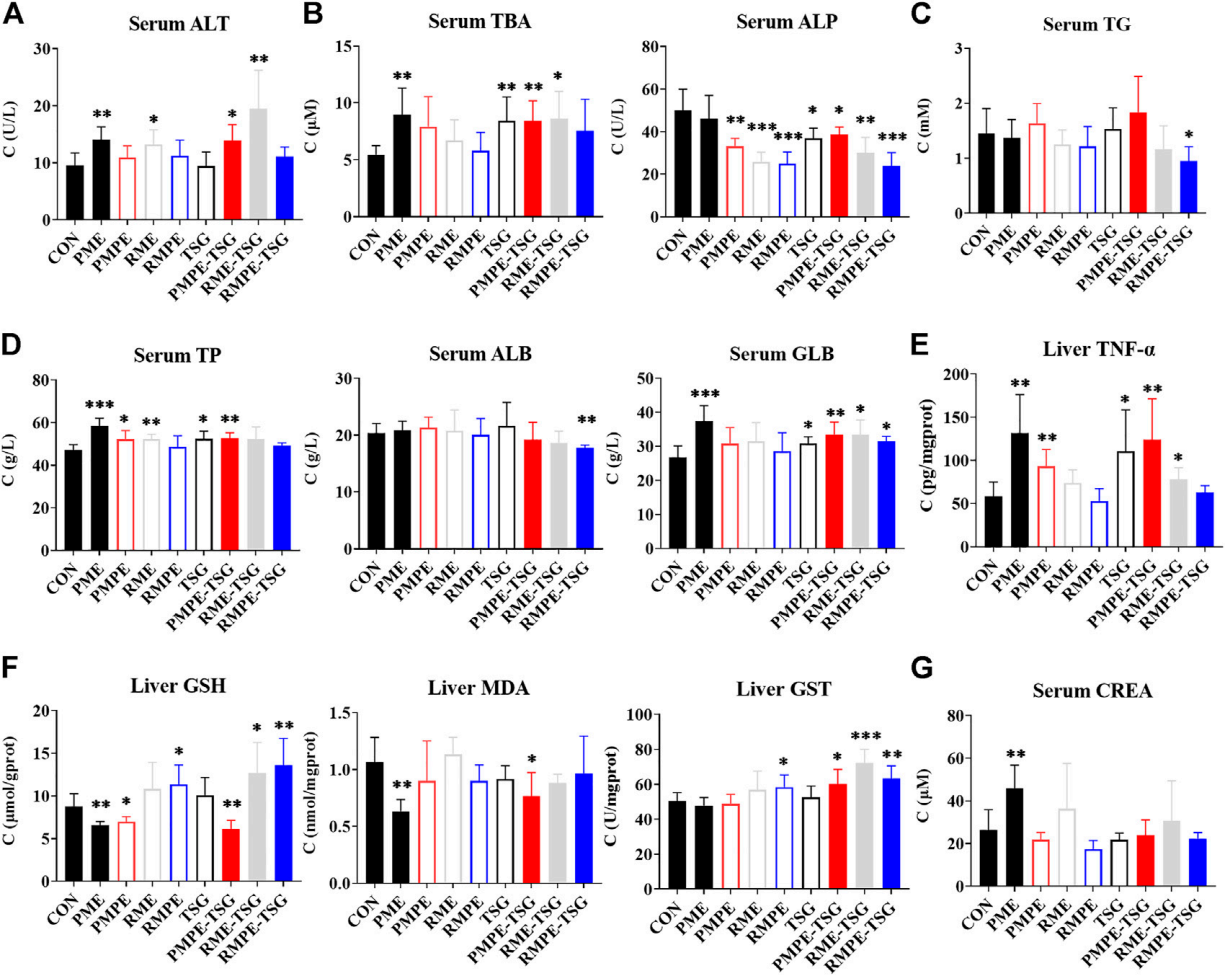
The serum and intrahepatic biochemical indexes of each group in the PM hepatotoxicity study. Data were expressed as mean ± SD (*n* = 6); Two-tailed independent Student’s *t*-test was conducted between each experimental group and the CON group. When *p* < 0.05, there was a statistical significance. *: *p* < 0.05; **: *p* < 0.01; ***: *p* < 0.001. **(A)** Hepatocyte membrane leakage index ALT, **(B)** Cholestasis index TBA and ALP, **(C)** Lipid metabolism index TG, **(D)** Protein synthesis function indexes TP and ALB and body inflammatory signaling GLB (equal to TP minus ALB), **(E)** Inflammation index TNF-α (determined by enzyme-linked immunosorbent assay), **(F)** Oxidative stress index reduced GSH, MDA (lipid peroxidation product malondialdehyde) and GST, **(G)** Renal excretion function index CREA.

The biochemical indexes in the serum and liver of all the groups in this PM hepatotoxicity study are shown in Figure 4. Different biochemical abnormalities were found in each experimental group compared with the control. As shown in Figures 4A–E, the serum GLB, ALT, TBA and hepatic TNF-α levels in the PME, PMPE-TSG and RME-TSG groups were increased significantly with reference to those in the CON group (*p* < 0.05). Whereas, the levels of hepatic TNF-α in the PMPE group, serum GLB, TBA and hepatic TNF-α in the TSG group, serum ALT in the RME group and serum GLB in the RMPE-TSG group were also significantly increased (*p* < 0.05). Obviously, more biochemical abnormalities were presented in the PME, PMPE-TSG and RME-TSG groups than in the other groups, suggesting much stronger hepatotoxicity. Furthermore, both serum TBA and alkaline phosphatase (ALP) levels are usually elevated due to cholestasis. However, except for the PME group, serum ALP levels (Figure 4B) were significantly decreased in almost all the experimental groups (*p* < 0.05), implying that the serum ALP could be affected by other factors.

The hepatic redox indexes, including GSH, MDA and GST were also determined (Figure 4F). Hepatic GSH levels in the PME, PMPE and PMPE-TSG groups were reduced significantly compared with the CON group (*p* < 0.05), indicating that the GSHs had been largely consumed. In contrary, hepatic GSH levels were markedly elevated in the RMPE, RME-TSG and RMPE-TSG groups (*p* < 0.05), displaying different oxidative stress states from those in PM extracts-containing groups. In the PME and PMPE-TSG groups, levels of hepatic MDA as a product of lipid peroxidation were also reduced (*p* < 0.05), implying that severe oxidative damage had not yet occurred. Meanwhile, levels of hepatic GST mediating GSH binding to toxic components were elevated significantly in the RMPE, PMPE-TSG, RME-TSG and RMPE-TSG groups (*p* < 0.05). These redox abnormalities suggested that GSH was involved in the metabolism of oxidizing components of these herbal extracts, causing the risk of GSH depletion.

As shown in Figure 4G, serum CREA level increased significantly in the PME group compared with the CON group (*p* < 0.01), and those in the RME and RME-TSG groups increased slightly as well, meaning that the renal excretion function declined in these groups. The measured biochemical values are listed in Supplementary Table S5.

### Tissue distributions of seven key characteristic components in mice following the toxicological evaluation

Herbal toxicity is closely associated with its main components’ exposure to target tissue. Hence, this part moved on to focus on tissue distribution (including liver, kidney, heart, spleen, lung, brain, stomach, large intestine and small intestine) in mice following the toxicological evaluation. Seven key components (TSG, EMG and five free anthraquinones, including rhein, emodin, chrysophanol, aloe-emodin and physcion) in mice tissues were determined to clarify the relationship between their exposure and toxicity in the target tissue by a validated HPLC-MS/MS method.

**FIGURE 5:**
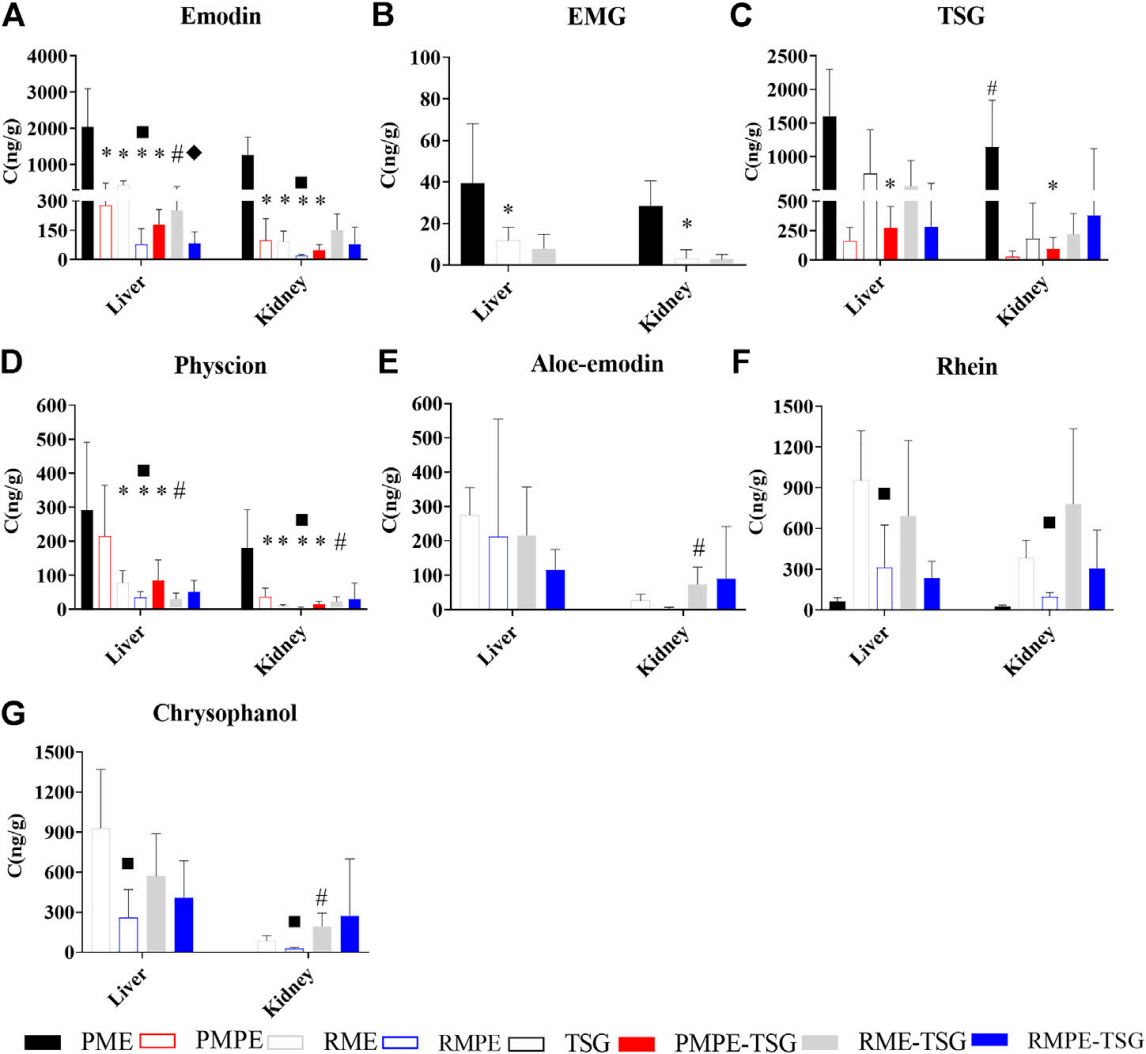
The contents of seven key components in mice liver and kidney tissues of each experimental group for the PM hepatotoxicity study. Data were expressed as mean ± SD (*n* = 6); Two-tailed independent Student’s *t*-test was conducted between two experimental groups. When *p* < 0.05, there was a statistical significance. *: *p* < 0.05, PMPE, RME, RMPE or PMPE-TSG group *versus* PME group; #, *p* < 0.05, PME or each combination group *versus* corresponding herbal extract-only group or TSG group; ■, *p* < 0.05, RMPE group *versus* RME group; ♦, *p* < 0.05, RMPE-TSG group *versus* RME-TSG group. **(A)** emodin; **(B)** EMG; **(C)** TSG; **(D)** Physcion; **(E)** Aloe-emodin; **(F)** Rhein; **(G)** Chrysophanol.

As illustrated in Figure 5, in PME, PMPE and three combination groups, both hepatic/renal emodin and TSG exposure levels were higher than EMG and physcion. In contrast, in RME, RMPE and their combination groups, the hepatic exposure levels of rhein and chrysophanol were higher than the other components. Rhein could be detected in the liver and kidney of the PME group, which was obviously bio-transformed from other anthraquinones.

Figure 5A displays the hepatic/renal emodin exposure. The hepatic emodin exposure levels are ranked as: PME > RME > PMPE ≈ RME-TSG > PMPE-TSG > RMPE > RMPE-TSG, while the renal ranked as: PME > RME-TSG > PMPE = RME > RMPE-TSG > PMPE-TSG > RMPE. Emodin in the raw herbal extracts-containing groups had notably higher hepatic/renal exposure levels than those in the corresponding concocted herbal extracts-containing groups, even though they have been designed with an equivalent emodin dose of 29 mg/kg. Moreover, after combination with TSG, TSG tended to decrease hepatic exposure levels of emodin while increasing its renal exposure levels (Figure 5A). This phenomenon was also partly manifested in the hepatic/renal exposure levels of physcion, aloe-emodin, rhein, and chrysophanol (Figures 5D–G).

Figure 5B shows the hepatic/renal EMG exposure. The hepatic/renal EMG could only be measured in the PME, RME and RME-TSG groups with higher EMG doses.

Figure 5C shows the hepatic/renal TSG exposure. The PMPE group (346 mg/kg) had lower hepatic/renal TSG exposure levels than the other TSG-containing groups (1,345 mg/kg). The PME group had much higher hepatic/renal TSG exposure levels than the other groups, suggesting a higher bioavailability of TSG in PME. Furthermore, in comparison with the TSG-only group, the hepatic/renal TSG exposure levels of the three combination groups had no significant changes.

The measured contents of these seven key components in each tissue (listed in Supplementary Tables S6–S12) showed that in addition to the high exposure levels in the liver and kidney, each component was mainly accumulated in the gastrointestinal tract of the mice for the multi-dose administration. Meanwhile, they all had lower exposure levels in the plasma than in the liver and kidney.

### Dose-exposure-response correlation analysis of the characteristic components in mice in this PM hepatotoxicity study

#### Dose-exposure correlation analysis of the characteristic components

Pearson correlation analysis using SPSS23 was performed on the 9 study groups between the dose levels of key components in the herbal extracts and the hepatic/renal exposure levels of seven key components in mice in this PM hepatotoxicity study. As shown in Table 3, the dose level of each anthraquinone glycoside in these groups had a positive correlation with the hepatic/renal exposure level of each corresponding free anthraquinone. The EMG (the main anthraquinone glycoside form of emodin glycosides) dose levels had high positive correlations with hepatic and renal emodin exposure levels (correlation coefficients >0.812, *p* < 0.05), and moderate correlations with hepatic and renal TSG and physcion exposure levels (correlation coefficients >0.540, *p* < 0.05). TSG dose levels showed no significant correlations with the hepatic exposure levels of anthraquinone components, but weak correlations with the renal emodin and physcion exposure levels.

**TABLE 3:** Pearson correlation coefficients between dose level of each characteristic component of PM and rhubarb and the hepatic and renal exposure (*n* = 6).

Component	Liver							Kidney						
	EM	TSG	EMG	PH	AE	RE	CH	EM	TSG	EMG	PH	AE	RE	CH
EM	0.287*	—	—	0.377**	0.305*	0.335*	0.345*	—	—	—	0.269*	—	0.297*	—
EMG	0.812**	0.604**	0.772**	0.540**	—	—	—	0.822**	0.568**	0.829**	0.695**	—	—	—
TSG	—	0.551**	—	—	—	—	—	0.310*	0.409**	0.272*	0.301*	—	—	—
PH	0.361**	—	0.315*	0.342*	0.354**	0.352**	0.374**	0.351**	—	0.321*	0.344*	—	0.312*	—
AE	—	−0.292*	—	−0.281*	0.627**	0.636**	0.700**	—	—	—	—	0.402**	0.579**	0.439**
RE	—	−0.297*	—	−0.281*	0.601**	0.579**	0.651**	—	—	—	—	0.394**	0.529**	0.436**
CH	—	−0.290*	—	−0.281*	0.634**	0.652**	0.713**	—	—	—	—	0.403**	0.594**	0.438**
EM-G	0.781**	0.575**	0.757**	0.505**	—	0.300*	—	0.785**	0.536**	0.797**	0.657**	—	—	—
PH-G	0.589**	0.367**	0.609**	0.371**	0.317*	0.538**	0.472**	0.560**	0.355**	0.578**	0.458**	—	0.465**	—
AE-G	—	—	—	—	0.583**	0.799**	0.784**	—	—	—	—	0.311*	0.714**	0.306*
RE-G	—	—	—	—	0.583**	0.799**	0.784**	—	—	—	—	0.311*	0.714**	0.306*
CH-G	—	—	—	—	0.616**	0.807**	0.805**	—	—	—	—	0.340*	0.723**	0.342*

*: *p* < 0.05; **: *p* < 0.01; ***: *p* < 0.001. EM, emodin; PH, physcion; AE, aloe-emodin; RE, rhein; CH, chrysophanol; EM-G, aglycones of hydrolyzed emodin glycosides; PH-G, aglycones of hydrolyzed physcion glycosides; AE-G, aglycones of hydrolyzed aloe-emodin glycosides; RE-G, aglycones of hydrolyzed rhein glycosides; CH-G, aglycones of hydrolyzed chrysophanol glycosides.

#### Exposure-response canonical correlation analysis of the characteristic components

In this study, CCA as a multivariate statistical model performed by SPSS23 was used to analyze the overall correlation between biochemical abnormalities of liver function (ALT, TBA, ALP, TG, ALB, GLB, TNF-α, GSH, MDA, GST, etc.) and the hepatic exposure of the seven key components (TSG, EMG, emodin, physcion, aloe emodin, rhein, and chrysophanol) in this PM hepatotoxicity study. Two sets of original variables are converted to several pairs of two dependent canonical variables by the CCA model. The first pair of canonical variables has the highest correlation. Each original variable, with an absolute value of canonical loading (the correlation coefficient between the original variable and the canonical variable) greater than 0.3, is selected as the variable with great influence on the overall variable (Wu et al., 2022).

The correlation coefficient of the first pair of canonical variables was 0.822 (*p* < 0.01, F was 1.937). Canonical loading results are shown in Table 4. The biochemical alterations in liver function were mainly represented by GLB, TNF-α, ALP, TBA, MDA, GSH and GST, while the alterations in hepatic exposure of seven key components were mainly represented by TSG, emodin, physcion, EMG and aloe-emodin.

**TABLE 4:** Canonical correlation coefficients between hepatic exposure and biochemical index in this PM hepatotoxicity study (*n* = 6).

Set	Variable	Standardized canonical correlation coefficients	Canonical loadings
Set 1	EML	−0.367	−0.794
	TSGL	−0.425	−0.831
	EMGL	0.009	−0.604
	PHL	−0.279	−0.728
	AEL	0.477	0.335
	REL	−0.616	0.124
	CHL	0.303	0.248
Set 2	ALT	−0.238	−0.278
	TBA	−0.189	−0.392
	ALP	−0.211	−0.456
	TG	0.295	−0.051
	ALB	−0.132	−0.064
	GLB	−0.581	−0.726
	TNF-α	−0.248	−0.560
	GSH	0.061	0.433
	MDA	0.145	0.495
	GST	0.360	0.310

#### Exposure-response pearson correlation analysis of the characteristic components

Pearson correlation analysis using SPSS23 was performed on the 9 study groups between the hepatic/renal exposure and the relevant biochemical abnormalities in this PM hepatotoxicity study. As shown in Table 5, the hepatic emodin exposure level had weak positive correlations with the serum GLB and hepatic TNF-α levels, and a weak negative correlation with the hepatic GSH level (all the absolute correlation coefficients >0.28, *p* < 0.05). The hepatic TSG, EMG and physcion exposure levels all had weak positive correlations with the serum TBA and GLB levels, and hepatic TNF-α level (all the correlation coefficients >0.32, *p* < 0.05). The hepatic aloe-emodin, emodin and chrysophanol exposure levels all had a weak positive correlation with the serum ALT level (all the correlation coefficients >0.30, *p* < 0.05). These biochemical abnormalities were associated with adverse effects on liver function.

**TABLE 5:** Pearson correlation coefficients between hepatic and renal exposure and biochemical index in this PM hepatotoxicity study (*n* = 6).

Tissue	Component	ALT	TBA	ALP	TG	ALB	TP	GLB	CREA	TNF-α	GSH	MDA	GST
Liver	EM	—	—	—	—	—	0.449**	0.352**	0.478**	0.442**	−0.276*	−0.353**	−0.280*
	TSG	—	0.321*	0.322*	—	—	0.558**	0.505**	0.443**	0.323*	—	−0.419**	—
	EMG	0.303*	0.328*	—	—	—	0.377**	0.317*	0.497**	0.300*	—	—	—
	PH	—	0.306*	—	—	—	0.495**	0.433**	0.336**	0.292*	−0.365**	−0.358**	−0.276*
	AE	0.297*	—	−0.434**	−0.310*	—	—	—	—	−0.305*	0.385**	—	0.288*
	RE	0.474**	—	−0.393**	−0.337*	—	—	—	—	—	0.380**	—	0.360**
	CH	0.316*	—	−0.471**	−0.362**	—	—	—	—	—	0.469**	—	0.316*
Kidney	EM	—	—	0.308*	—	—	0.553**	0.479**	0.552**	0.398**	—	−0.373**	—
	TSG	—	—	—	—	—	0.361**	0.381**	0.406**	0.282**	—	—	—
	EMG	—	—	0.317*	—	—	0.523**	0.469**	0.454**	0.340**	—	−0.355**	−0.273*
	PH	—	—	—	—	—	0.495**	0.455**	0.477**	0.383**	—	−0.274*	—
	AE	—	—	−0.311*	−0.317*	—	—	—	—	—	0.508**	0.309*	0.425**
	RE	0.528**	—	−0.345*	−0.391**	—	—	—	—	—	0.552**	—	0.495**
	CH	—	—	−0.331*	−0.335*	—	—	—	—	—	0.547**	—	0.453**

*: *p* < 0.05; **: *p* < 0.01; ***: *p* < 0.001. EM: emodin, PH: physcion, AE: aloe-emodin, RE: rhein, CH: chrysophanol.

Meanwhile, the hepatic emodin, physcion and TSG exposure levels all had a weak negative correlation with the hepatic MDA level (absolute correlation coefficients >0.35, *p* < 0.05). The hepatic aloe-emodin, emodin and chrysophanol exposure levels had weak negative correlations with the serum TG and ALP levels (all the correlation coefficients >0.31, *p* < 0.05), and weak positive correlations with the hepatic GSH and GST levels (all the correlation coefficients >0.29, *p* < 0.05). These biochemical abnormalities all reflected the hepatic protection effect, instead.

Finally, there was a moderate positive correlation between renal TSG, EMG, emodin and physcion exposure levels and serum CREA level (correlation coefficients >0.41, *p* < 0.05).

### Hepatotoxicity of three key components of PM ethanol extract and their combinations *in Vitro*

**FIGURE 6:**
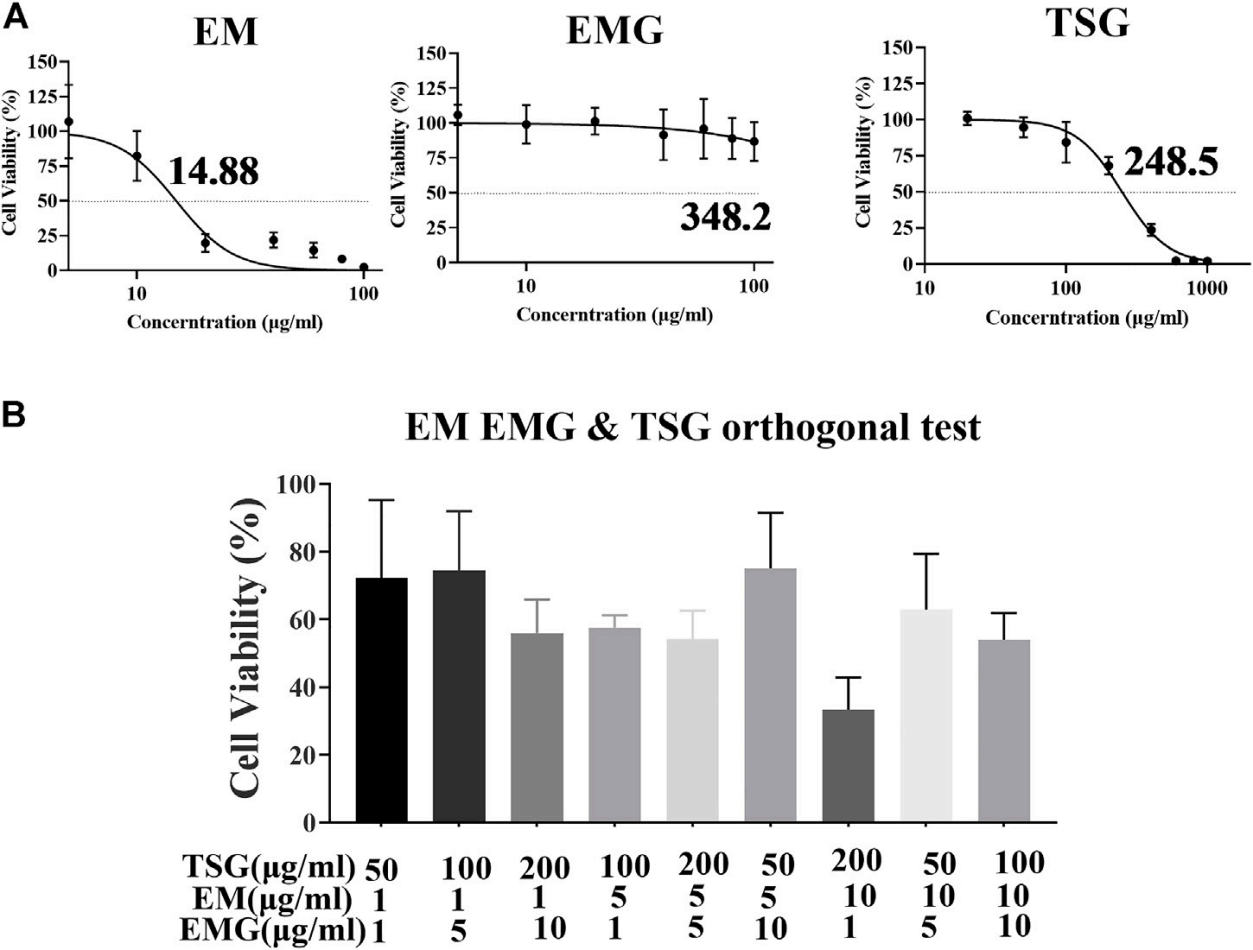
Orthogonal experiment results of TSG, EM and EMG combination in L02 cell line. Data were expressed as mean ± SD (n = 3 independent replicates). **(A)** Concentration-cell viability curves for emodin at 1–100 μg/ml, EMG at 1–100 μg/ml, and TSG at 20–1000 μg/ml. **(B)** Cell viability after the treatment of nine orthogonal combinations of TSG, EMG and emodin at high, medium and low concentration levels for 48 h.

As shown in Figure 6A, the IC50 of emodin, EMG and TSG were 14.88, >100 (fitting value was 348.2) and 248.5 μg/ml, respectively. This indicated that emodin was far more hepatotoxic than the other two substances *in vitro*. As shown in Figure 6B, the cell inhibitory activity was enhanced after combination compared with a single component. According to Table 6, the CI values of the three in different combinations were generally less than 0.9, meaning a mild additive or synergistic effect. The orthogonal test results (Table 6) demonstrated that the inhibitory effect was mainly from TSG and emodin according to the range analysis and one-way ANOVA of each level of the combinations of the three key components. TSG/emodin ratio was positively correlated with L02 cell inhibition according to one previous report (Yu et al., 2011). This study further elucidated the synergistic cell inhibitory effect of emodin and TSG.

**TABLE 6:** Orthogonal test results, range and one-way ANOVA analysis, and CI for three characteristic components of PM in L02 cell (*n* = 3 independent repeated experiments).

Experimental number	Factor				Cell viability (%)	CI
	Emodin	EMG	TSG	Blank		
N1	1	1	50	1	72.2	0.40
N2	1	5	100	2	74.5	0.80
N3	1	10	200	3	55.9	1.13
N4	5	1	100	3	57.6	0.75
N5	5	5	200	1	54.2	1.24
N6	5	10	50	2	75.1	0.70
N7	10	1	200	2	33.4	1.04
N8	10	5	50	3	62.9	0.76
N9	10	10	100	1	54.1	0.94
K1	67.5	54.4	70.1	60.2	—	—
K2	62.7	63.9	62.0	61.0	—	—
K3	55.9	61.7	47.8	58.8	—	—
R value	11.7	9.4	22.3	2.2	—	—
Sum of Squares	478.176	147.602	760.909	7.402	—	—
df	2	2	2	2	—	—
F value	64.6	19.9	102.8	—	—	—
*p* value	0.015	0.048	0.01	—	—	—

## Discussion

Each part of PM extracts extracted by various polarities of solvents has all been shown to have certain hepatotoxicity in rats (Zhang et al., 2018). However, due to the component complexity of each PM extract part, it is still difficult to elucidate the basis of PM hepatotoxic substances. Because rhubarb, an herb of the Polygonaceae family like PM, has rich similar free anthraquinones and anthraquinone glycosides, but has not been reported with hepatotoxicity in humans, so its toxicological comparison with PM extract and combined hepatotoxic effect with TSG (major and unique characteristic component in PM) in mice would disclose the role of the different components of PM and TSG in PM-induced hepatotoxicity.

The results of the toxicological evaluation (Figures 3, 4) demonstrated that oral administration of PME (8.3 g/kg) for 28 days in mice produced hepatotoxicity, characterized by biochemical abnormalities of ALT, TBA and GLB in the serum, and TNF-α and GSH in the liver, as well as significant histological alterations in the liver. In rodent species, PME has also been shown to cause redox imbalance, nutrient and bile acid metabolism disorders and liver inflammation (Li Y. X. et al., 2017; Lin et al., 2017; Ruan et al., 2019). However, in the RME group (10.4 g/kg), no severe liver damage was recorded, except for the elevated level of serum ALT, indicating that RME had a weaker impact on liver function than PME. Nevertheless, total rhubarb extract (extract combination of 90% ethanol and water) still possesses the potential to cause sub-chronic liver toxicity in rats (Wang et al., 2011). In addition, both PME and RME also caused body weight loss along with diarrhea, and mild nephrotoxicity in mice (Figures 3A,C). Both PMPE (6.5 g/kg) and RMPE (4.8 g/kg) had an equivalent emodin dose of 29 mg/kg to PME and RME. They could also affect several biochemical indexes, but caused no liver lesions, diarrhea and renal pathological changes. In comparison with the corresponding concocted herbs, anthraquinone glycoside doses in raw PM or rhubarb herbal extracts increased, so did the TSG dose in raw PM herbal extract, which may be the reason why raw herbs are more toxic.

Similarly, TSG with the equivalent TSG dose of 1,345 mg/kg to PME could markedly elevate the levels of TP, GLB and TBA in the serum, and TNF-α in the liver compared with the control, although no liver tissue lesions were observed. In contrast, the combination of TSG with PMPE (or with RME) caused biochemical and histopathological liver injuries in mice like PME. Therefore, it can be concluded that TSG is one of the essential components in PM-induced liver injury.

According to the tissue distribution study (Figure 5 and Supplementary Table S6–S12), seven key components had higher exposure levels in the liver and kidney than in the serum due to their stronger tissue affinity, which was consistent with the literature (Li et al., 2021). In the mice receiving PME, PMPE-TSG and RME-TSG, the hepatic exposure levels of TSG and emodin were much higher than those of EMG and physcion. A toxicokinetic study has shown that an overdose of PM extract can lead to a gradual increase of emodin exposure in rats along with the dosing time (Ma et al., 2015). Considering the reported emodin potential effects of cytotoxicity, cholestasis and GSH depletion, emodin is inferred to be another potential substance of PM hepatotoxicity.

Although each containing extract had the equivalent emodin dose, these groups still had different exposure levels of emodin. Dose-exposure correlation analysis (Table 3) revealed that the hepatic/renal emodin exposure in mice had strong positive correlations with EMG dose level. According to previous reports, emodin itself has low bioavailability (Shia et al., 2010), and the bioavailability of emodin in the plasma of the rats treated with PM extract is higher than that of PMP extract (Zhang et al., 2013). This study confirmed that the *in vivo* exposure of emodin was mainly affected by EMG. In addition, a pre-treatment with TSG in rats may significantly enhance the plasma emodin exposure levels by inhibiting UDP-glucuronosyltransferases 1A8 (Ma et al., 2013). However, hepatic emodin exposure level in mice was slightly reduced after co-administration in this study. This could be attributed to the increased Phase I metabolism of emodin *via* the CYP1A2 expression induced by TSG (Xing et al., 2019). Therefore, the emodin exposure was mainly affected by EMG rather than TSG.

In rhubarb extract-containing groups, the hepatic exposure levels of rhein and chrysophanol were higher than emodin. Rhein is also highly hepatotoxic *in vitro* (Bounda et al., 2015). Thus, rhein could not be excluded as a potential substance basis for RME-TSG-induced liver damage in mice. The RME-TSG-induced hepatotoxicity was also partly attributed to its anthraquinone glycosides. In contrary, RMPE-TSG did not cause liver damage in mice because its fewer anthraquinone glycosides than RME-TSG could not increase the hepatic exposure levels of emodin and rhein.

According to the CCA results for exposure-response (Table 4), the biochemical abnormalities of liver function in each group were mainly reflected in GLB, TNF-α, ALP, TBA, MDA, GSH, GST, and were mainly affected by the changes of hepatic TSG, emodin, physcion, EMG, and aloe-emodin. Moreover, Pearson correlation analysis (Table 5) between two univariates distinguished the related components to different biochemical abnormalities. Firstly, serum GLB and hepatic TNF-α levels had weak positive correlations with hepatic emodin, TSG, EMG, and physcion exposure levels, suggesting that these four components of PM may induce liver inflammation. And these groups showed a significant increase of neutrophils in the peripheral blood cells (Supplementary Figure S4), implying an establishment of body inflammation. Interestingly, each experimental group had a markedly reduced thymus index compared with the control (Supplementary Figure S5), which may aggravate liver inflammation by affecting the immune homeostasis. Secondly, serum TBA levels had weak positive correlations with hepatic TSG, EMG and physcion levels, but serum ALP level was significantly decreased in each group, suggesting that these three components may induce cholestasis but serum ALP could not be used to indicate the degree of cholestasis. Thirdly, hepatic emodin exposure level had a weak negative correlation with hepatic GSH level, but hepatic TSG, emodin and physcion exposure levels displayed negative correlations with hepatic MDA levels, reflecting the redox two-way effects induced by them. This two-way effect may cause nonlinear hepatotoxicity of PM, so that a low dose of PM ethanol extract by oral gavage for 28 days has caused stronger hepatotoxicity in mice than a medium dose (Ruan et al., 2019). In addition, the hepatic exposure levels of aloe-emodin, rhein and chrysophanol had weak positive correlations with the hepatic GSH and GST levels, but weak negative correlations with serum TG levels, which implied a hepatic protection effect and provided one explanation why the hepatotoxicity of RME was lower than that of PME.

Emodin and TSG in PM were related with the biochemical abnormalities of liver function in this study, and had the higher exposure levels than EMG and physcion. EMG is the main anthraquinone glycoside component in PM, and it enhances emodin exposure. Thus, the inhibitory and synergistic inhibitory effects on L02 cells *in vitro* of the three components were measured. As a result, emodin and TSG were the two predominant substances of the three, which had mild additive or synergistic cell inhibitory effects in the orthogonal combination test (Table 6). Therefore, the co-hepatotoxic effect of high exposure levels of emodin and TSG was one of the mechanisms of PM hepatotoxicity, and EMG played an indirect role through *in vivo* biotransformation to emodin.

Obviously, PM hepatotoxicity is associated with multiple components with effecacy-toxicity effects on the liver and their interactions. Therefore, one should avoid using PM at an overdose and taking the anthraquinone-containing drugs or food with PM products simultaneously. The integrative analysis of various biochemical abnormalities, especially inflammation indexes, and liver biopsy was necessary for the diagnosis of PM-induced hepatotoxicity. The plasma TSG and emodin should be monitored over a long treatment course.

## Conclusion

In summary, this study creatively revealed the different contributions of TSG, EMG and emodin to PM-induced hepatotoxicity through comparative studies of toxicology and tissue distribution in mice using rhubarb as a reference. PM and rhubarb toxicological differences primarily originated from whether they contained TSG and more EMG contents. The coexistence of TSG and emodin with high hepatic exposure were the direct substances basis of PM hepatotoxicity causing hepatocyte toxicity, cholestasis and liver inflammation. EMG was the leading factor in enhancing the emodin exposure in the liver, playing an indirect hepatotoxic effect. This study provided toxicological and pharmacokinetic evidences for the rational clinical application of PM and rhubarb, and a novel method for the comparative study of similar herbal medicines.

## Data Availability

The original contributions presented in the study are included in the article/Supplementary Material, further inquiries can be directed to the corresponding author.

## Glossary

**AE**: Aloe-emodin
**AE-G**: Aglycones of hydrolyzed aloe-emodin glycosides
**ALB**: Albumin
**ALP**: Alkaline phosphatase
**ALT**: Alanine aminotransferase
**CCK-8**: Cell counting kit-8
**CH**: Chrysophanol
**CH-G**: Aglycones of hydrolyzed chrysophanol glycosides
**CON**: Control
**CREA**: Creatinine
**EM**: Emodin
**EM-G**: Aglycones of hydrolyzed emodin glycosides
**EMG**: Emodin-8-*O-β*-D-glucoside
**GLB**: Globulin
**GSH**: Glutathione
**GST**: Glutathione transferase
**IS**: Internal standard
**LLOQ**: Lower limit of quantitation
**MDA**: Malondialdehyde
**PH**: Physcion
**PH-G**: Aglycones of hydrolyzed physcion glycosides
**PM**: *Polygoni Multiflori Radix*
**PME**: PM ethanol-extract
**PMP**: *Polygoni Multiflori Radix Praeparata*
**PMPE**: PMP ethanol-extract
**PMPE-TSG**: PMPE-TSG combination
**QC**: Quality control
**RE**: Rhein
**RE-G**: Aglycones of hydrolyzed rhein glycosides
RME: Rhubarb ethanol-extract
**RME-TSG**: RME-TSG combination
**RMPE**: Steamed rhubarb ethanol-extract
**RMPE-TSG**: RMPE-TSG combination
**TBA**: Total bile acids
**TG**: Triglycerides
**TNF-α**: Tumor necrosis factor -α
**TP**: Total protein
**TSG**: 2,3,5,4′-Tetrahydroxystilbene-2-*O-β*-D-glucoside
