# Supplementary material for: Hepatotoxic metabolites in Polygoni Multiflori Radix— Comparative toxicology in mice
Source: Front Pharmacol. 2022 Oct 11;13:1007284. doi: 10.3389/fphar.2022.1007284 (PMC9592908; doi:10.3389/fphar.2022.1007284)
Supplement: Supplementary file 1 [file DataSheet1.docx]

Supplementary Material

# HPLC-UV Analytical Method for the Determination of the Key Characteristic Components of PME, PMPE, RME or RMPE

**Table S1** lists the HPLC-UV analytical method for the determination of key characteristic components (TSG, EMG, fiver free anthraquinones and five anthraquinone glycosides) of four herbal extracts. The herbal extracts were analyzed using reversed-phase high performance liquid chromatography (HPLC). The analysis was performed on an Agilent 1100 high performance liquid system (Agilent Technologies, California, USA) with an ultraviolet wavelength (UV) detector, and the separation was carried out on a Hedera ODS3 C18 column (4.6 × 250 mm, 5 μm) at 35℃ with mobile phase A (0.1% phosphoric acid aqueous solution) and mobile phase B (methanol) in linear gradient elution mode (A:B): 0 min (70:30) → 20 min (15:85) → 36 min (15:85) → 37 min (70:30) → 40 min (70:30). The flow rate was set at 1.0 mL/min and the detection wavelength was set at 275nm. The injection volume was 10 μL.

# Method Validation for the Tissue Distribution Study Following the Toxicological Evaluation in This PM Hepatotoxicity Study

The specificity, linear and range, accuracy and precision, matrix effect, extraction recovery, and sample stability of seven key characteristic components (TSG, EMG, emodin, physcion, aloe-emodin, rhein and chrysophanol) in mice tissues were investigated according to the ICH bioanalysis guideline. Their chemical structure and MS/MS spectrum information are shown in **Figure S1**. As shown in **Figure S2**, endogenous substances in the liver did not interfere with their determination. As listed in **Table S2**, the regression equations for the calibration curves (Weighting factor 1/X^2^) and LLOQs of these seven components. It was described that the correlation coefficient of each component was not less than 0.99 with good linearity. The calibration curves of the seven key components in the liver homogenate are shown in **Figure S3**. The LLOQs of TSG, EMG, rhein, emodin, chrysophanol, aloe-emodin, and physcion were 0.4, 0.1, 0.3, 0.1, 0.2, 0.2, and 0.2 ng/mL, respectively. As listed in **Table S3**, the relative standard deviations of the accuracy and the intra-batch and inter-batch precision, matrix effect and extraction recovery for this analytical method of each component at four QC concentrations were all within ± 15%. As listed in **Table S4**, the component stabilities in the liver homogenate samples were good whenever the samples were placed on the test bench at room temperature, the processed samples were placed in the autosampler tray for 24 hours at 15°C, the samples were under freezing and thawing conditions, or at long-term low temperature storage at −80°C.

# Biochemical Index Values Determined in this PM Hepatotoxicity Study

The measured biochemical values for each study group after 28 days of oral administration are listed in **Table S5**.

# Tissue Distribution Results Following the Toxicological Evaluation in This PM Hepatotoxicity Study

As listed in **Tables S6** to **S12**, the seven key components mainly had a high concentration in the gastrointestinal tract. Unexpectedly, aloe-emodin, rhein, and chrysophanol, which were specific to rhubarb extract, were detected in the mice gastrointestinal tract of the PME group and a little amount of rhein, obviously biotransformed through other anthraquinones, was also found in the liver and kidney of this group. Except for this, all of the detectable key components were mainly present in the liver and kidney tissues, and all but rhein were detected in the brain.

# Hematological Examination for the Peripheral Blood Cells in This PM Hepatotoxicity Study

Hematological examination was used to investigate the effect on peripheral blood cells after 28 days of oral administration. As seen in **Figure S4**, it was shown that NEU was increased more significantly in the PME, RMPE, PMPE-TSG, RME-TSG, and RMPE-TSG groups than in the CON group (*p* < 0.05). The WBC in the PME, PMPE, TSG and PMPE-TSG groups was also significantly increased (*p* < 0.01). High levels of neutrophils (NEU) and white blood cells (WBC) are generally associated with inflammation. The lymphocyte (LYM) in the RME-TSG group was significantly reduced (*p* < 0.05), which could be linked with cellular immunosuppression.

# Organ Index Determination in This PM Hepatotoxicity Study

The determined organ indexes of the liver, kidney, spleen and thymus in mice after 28 days of oral administration are presented in **Figure S5**. The damage often leads to tissue inflammation and swelling to increase the organ index, but it can also weaken the physiological function of the organs to reduce the organ index. It was shown that the liver indexes of the PMPE, TSG, and PMPE-TSG groups were significantly lower than those of the CON group (*p* < 0.01). Since it was impossible to induce organ atrophy for faster growth of mice in the PMPE group, the liver index reduction of these groups could only be attributed to the metabolic nutrients’ imbalance. The spleen and thymus are peripheral and central immune organs, respectively. The former is involved in humoral immunity, while the latter is responsible for cellular immunity and immune homeostasis. The spleen indexes of the PMPE, TSG, and PMPE-TSG groups were decreased significantly (*p* < 0.05), which suggested the inhibited spleen function, while the spleen indexes of the RME and RME-TSG groups were increased (*p* < 0.05), which indicated the enhanced humoral immune function of the spleen owing to body inflammation. In addition, thymus indexes for all the groups were significantly reduced (*p* < 0.05), which implied that both these herbal extracts and TSG had cellular immunosuppressive effects.


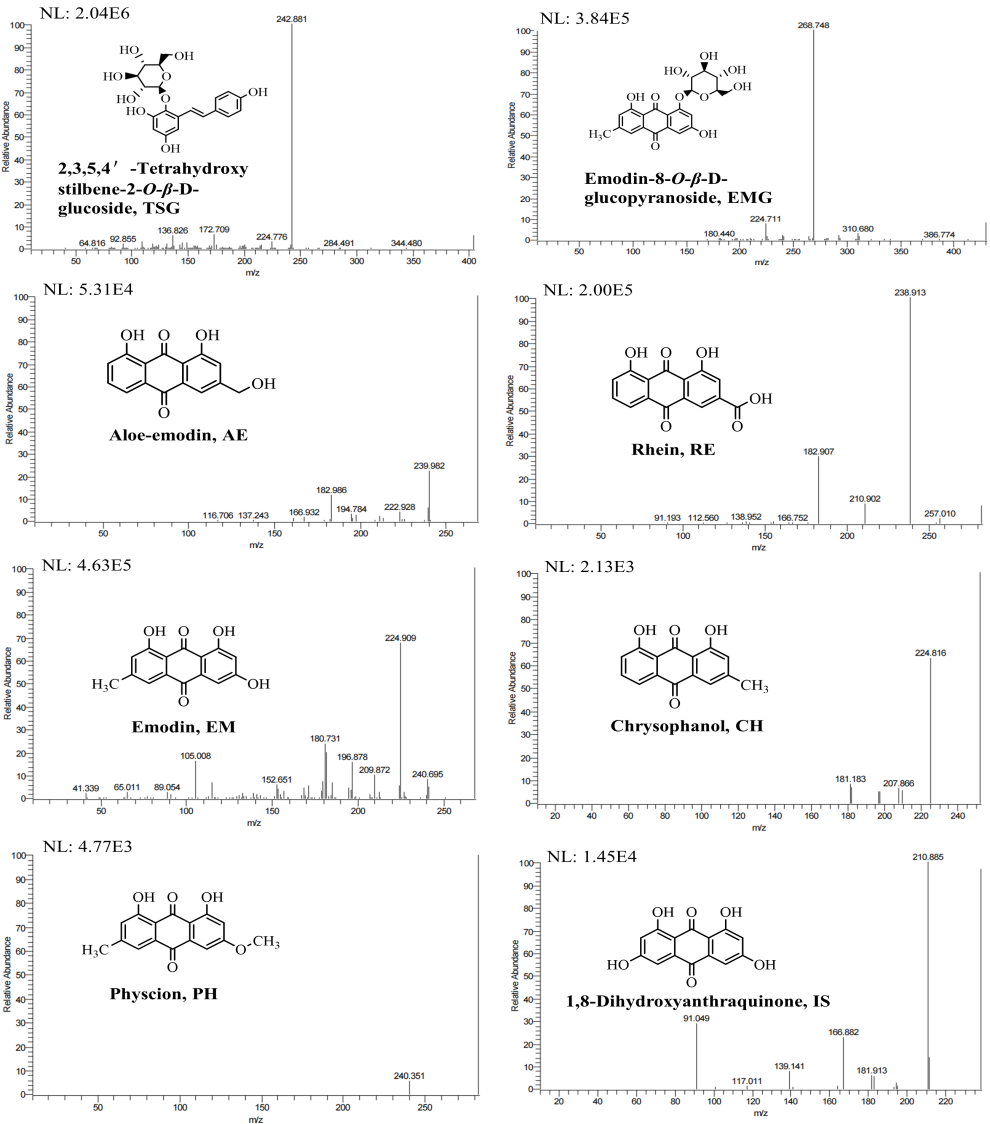


**FIGURE** **S1** The chemical structures and MS/MS spectrums of seven key components determined in mice tissues for PM hepatotoxicity study.

The MRM transitions of TSG, EMG and five free anthraquinones and IS were *m*/*z* 405.00 @22 eV → 242.89 for TSG; *m*/*z* 431.07 @29 eV → 268.88 for EMG; *m*/*z* 269.10 @23 eV → 239.87 for AE; *m*/*z* 283.10 @30 eV → 182.90 for RE; *m*/*z* 269.00 @26 eV → 224.88 for EM; *m*/*z* 253.00 @27 eV →224.80 for CH; *m*/*z* 283.20 @25 eV →240.34 for PH and *m*/*z* 239.00 @28 eV →210.97 for IS.

**
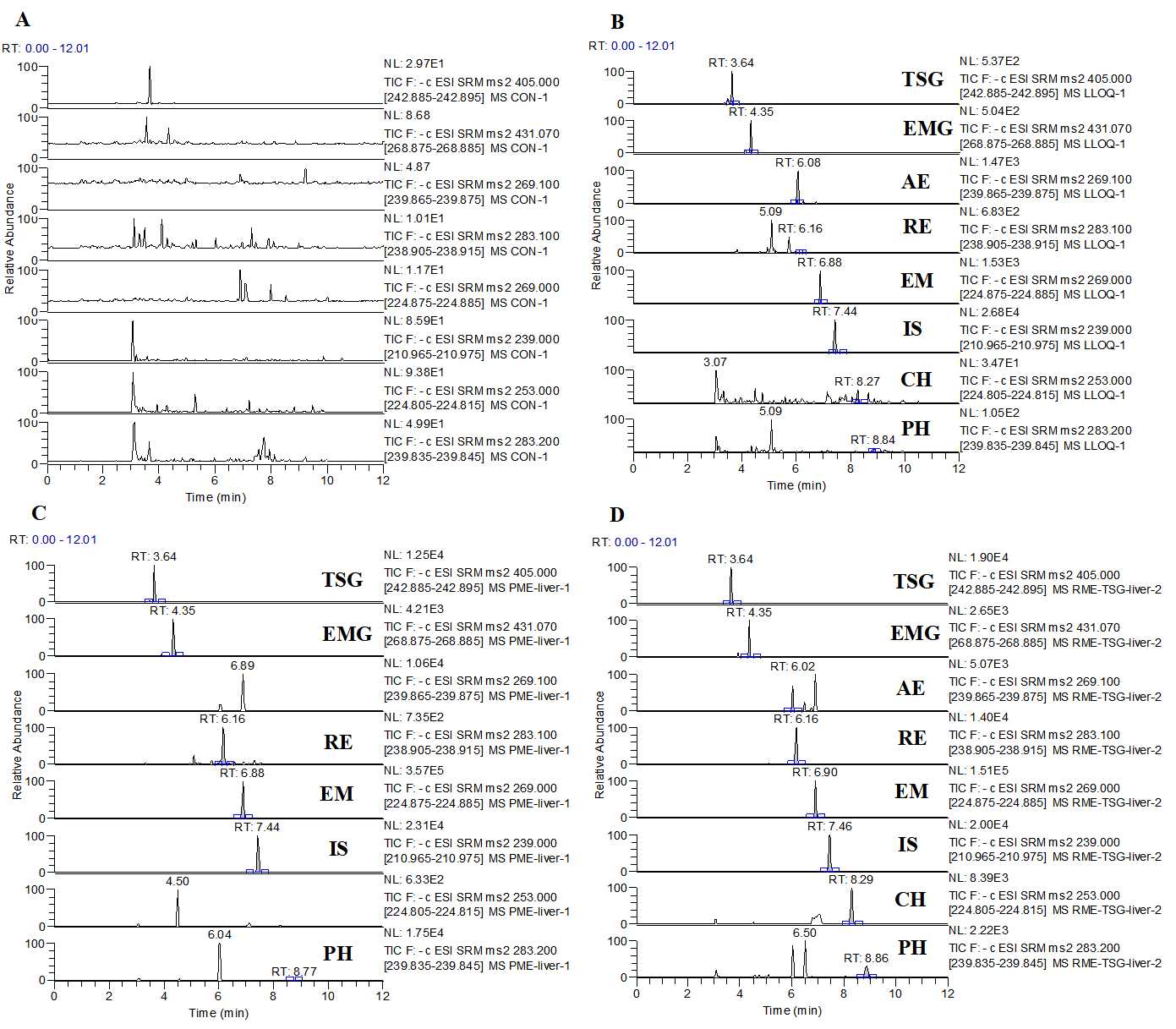
FIGURE** **S2** LC-MS/MS chromatograms of seven key components in mice liver homogenates for PM hepatotoxicity study.

(A) a blank liver sample; (B) a blank liver sample spiked with 0.4 ng/mL TSG, 0.1 ng/mL EMG, 0.2 ng/mL AE, 0.3 ng/mL RE, 0.1 ng/mL EM, 0.2 ng/mL CH and 0.2 ng/mL PH; (C) a liver sample after oral gavage of PME for 28 days containing 189 ng/mL TSG, 6.6 ng/mL EMG, 781 ng/mL EM, 22.7 ng/mL RE and 10.6 ng/mL PH; (D) a liver sample after oral gavage of RME-TSG for 28 days containing 38.5 ng/mL TSG, 2.0 ng/mL EMG, 62.7 ng/mL AE, 141 ng/mL RE, 73.6 ng/mL EM, 84.6 ng/mL CH and 2.3 ng/mL PH.


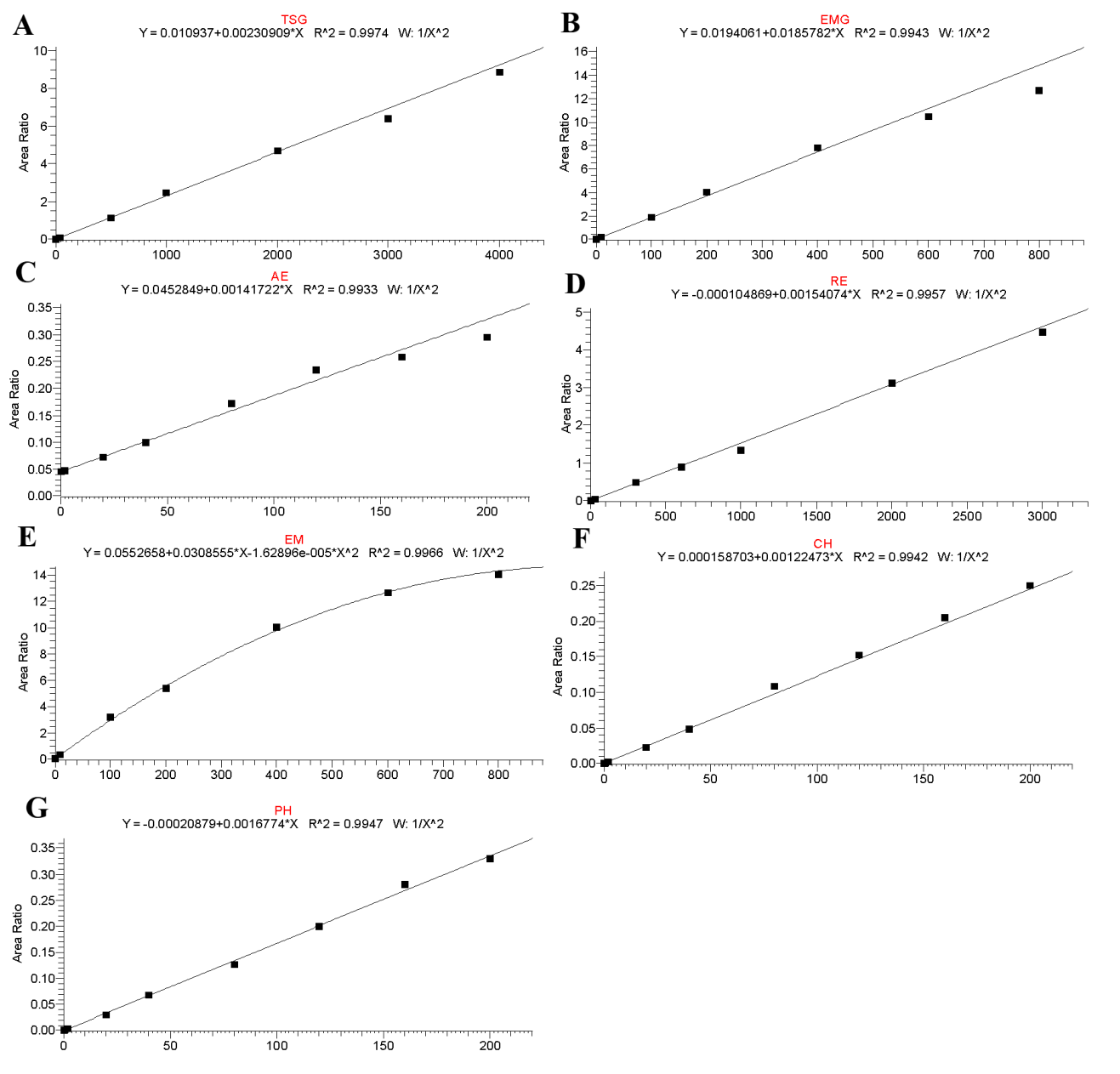


**FIGURE S3** Calibration curves of seven key components in mice liver homogenates (Weighting factor 1/X^2^) for PM hepatotoxicity study.

The linear ranges were 0.4-4,000 ng/mL for TSG; 0.3-3,000 ng/mL for rhein; 0.1-800 ng/mL for emodin and EMG; 0.2-200 ng/mL for aloe-emodin, chrysophanol and physcion.


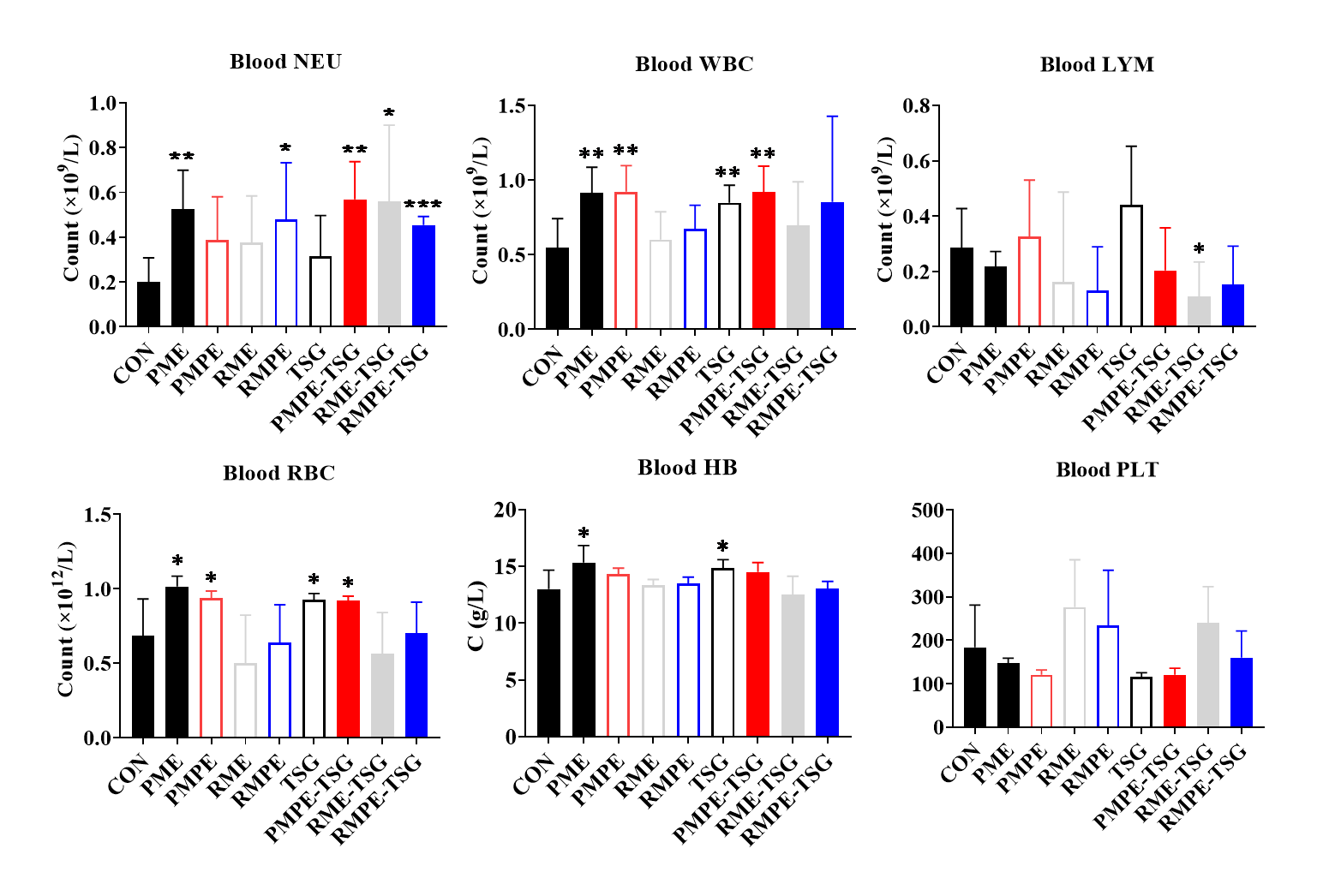


**FIGURE S4** The determined peripheral blood cells routine indexes in all groups for PM hepatotoxicity study.

Data were expressed as mean ± SD (n = 6); Two-tailed independent Student’s *t*-test was conducted between each experimental group and the CON group. When *p* < 0.05, there was a statistical significance. *: *p* < 0.05; **: *p*<0.01; ***: *p*<0.001.


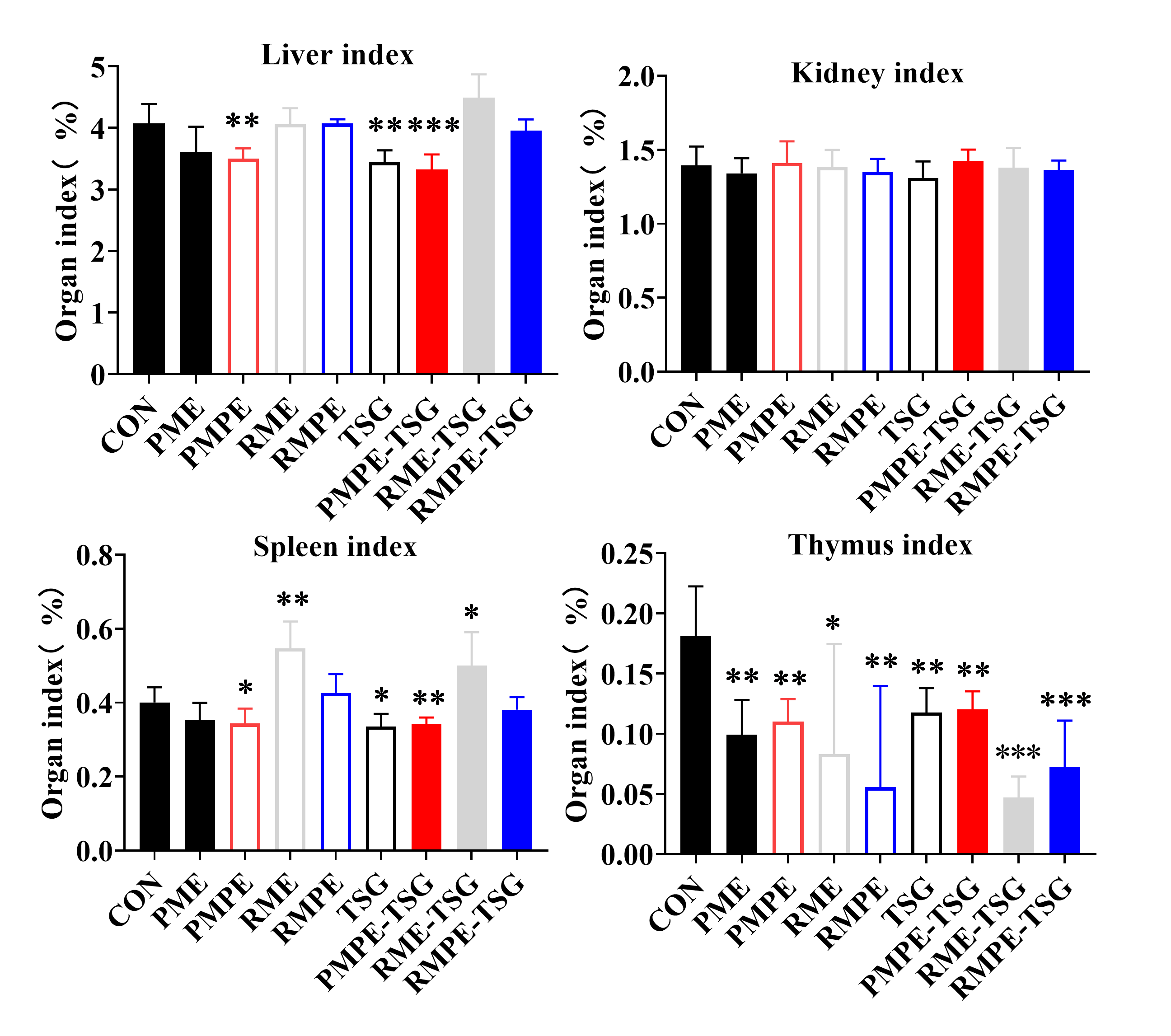


**FIGURE S5** The determined organ indexes of liver, kidney, spleen and thymus in all groups for PM hepatotoxicity study.

Data were expressed as mean ± SD (n = 6); Two-tailed independent Student’s *t*-test was conducted between each experimental group and the CON group. When *p* < 0.05, there was a statistical significance. *: *p* < 0.05; **: *p*<0.01; ***: *p*<0.001.

| **Item** | **Conditions** |
| --- | --- |
| High performance liquid system | Agilent 1100 |
| Column | Hedera ODS3 C18 column (4.6 × 250 mm, 5 μm) |
| Column temperature | 35℃ |
| Mobile phase | Mobile phase A (0.1% phosphoric acid aqueous solution) and mobile phase B (methanol) |
| Linear gradient elution mode (A:B) | 0 min (70:30) → 20 min (15:85) → 36 min (15:85) → 37 min (70:30) → 40 min (70:30). |
| Flow rate | 1.0 mL/min |
| Detection wavelength | 275nm |
| Injection volume | 10 μL |

**TABLE S1** HPLC-UV method for herbal extracts analysis for PM hepatotoxicity study.

| **Analyte** | **Regression equations** | **Coefficient (r^2^)** | **Range (ng/mL)** | **LLOQ (ng/mL)** | **RSD**  **(%)** | **RE**  **(%)** |
| --- | --- | --- | --- | --- | --- | --- |
| TSG | Y = 0.0109+0.00231*X | 0.9974 | 0.4-4,000 | 0.4 | 8.36 | −3.86 |
| EMG | Y = 0.0194+0.0186*X | 0.9943 | 0.1-800 | 0.1 | 7.43 | 5.31 |
| AE | Y = 0.0453+0.00142*X | 0.9933 | 0.2-200 | 0.2 | 6.9 | 4.14 |
| RE | Y = −0.000105+0.00154*X | 0.9957 | 0.3-3,000 | 0.3 | 7.31 | 0.66 |
| EM | Y = 0.0553+0.0309*X−1.63e−005*X^2 | 0.9966 | 0.1-800 | 0.1 | 3.35 | −1.67 |
| CH | Y = 0.000159+0.00122*X | 0.9942 | 0.2-200 | 0.2 | 14.45 | 2.68 |
| PH | Y = −0.000209+0.00168*X | 0.9947 | 0.2-200 | 0.2 | 6.17 | 4.21 |

**TABLE S2** Calibration curves (Weighting factor: 1/X^2^) and LLOQ for seven key components in mice liver homogenates for PM hepatotoxicity study (*n* = 6).

**TABLE S3** Summary of method validation results for seven key components in mice liver homogenates for PM hepatotoxicity study (*n* = 6).

| **Analyte** | **Concentration (ng/mL)** | **Intra-batch** | | **Inter-batch** | | **Recovery (%)** | | **Matrix effect (%)** | |
| --- | --- | --- | --- | --- | --- | --- | --- | --- | --- |
|  |  | **RSD (%)** | **RE (%)** | **RSD (%)** | **RE (%)** | **Mean±SD** | **RSD** | **Mean±SD** | **RSD** |
| TSG | 1 | 9.14 | 1.37 | 7.47 | 2.35 | 113±6 | 5.45 | 190±8 | 4.46 |
|  | 10 | 11.6 | 0.32 | 8.69 | −0.15 | 100±5 | 4.72 | 200±14 | 6.91 |
|  | 1000 | 11.0 | −2.53 | 7.88 | 1.52 | 105±4 | 3.61 | 230±6 | 2.39 |
|  | 3200 | 5.81 | −0.55 | 6.03 | 3.54 | 103±6 | 5.81 | 324±15 | 4.62 |
| EMG | 0.25 | 9.73 | 3.55 | 9.07 | 1.71 | 95.7±8.9 | 9.33 | 103±5 | 5.14 |
|  | 2.5 | 7.84 | 7.48 | 5.35 | 7.72 | 101±4 | 4.39 | 95.7±3.8 | 3.97 |
|  | 200 | 8.96 | 1.83 | 6.27 | 7.77 | 104±1 | 0.92 | 96.6±3.8 | 3.9 |
|  | 640 | 9.02 | 3.89 | 6.96 | 5.54 | 103±6 | 5.66 | 95.4±8.8 | 9.25 |
| AE | 0.5 | 11.5 | −3.91 | 9.6 | −3.14 | 90.4±9.0 | 9.96 | 102±5 | 4.67 |
|  | 5 | 10.4 | 1.38 | 8.61 | −2.29 | 94.2±9.7 | 10.4 | 99.3±6.4 | 6.4 |
|  | 80 | 11.3 | −5.36 | 9.03 | −4.97 | 92.2±5.4 | 5.83 | 90.3±8.9 | 9.83 |
|  | 160 | 9.46 | −2.35 | 8.64 | −0.96 | 102±2.7 | 2.69 | 93.1±9.4 | 10.1 |
| RE | 0.75 | 7.26 | 6.98 | 6.69 | 3.32 | 96.8±6.5 | 6.71 | 102±9 | 8.57 |
|  | 7.5 | 7.1 | 1.71 | 6.28 | −0.9 | 92.2±5.7 | 6.19 | 102±9 | 8.48 |
|  | 600 | 9.2 | 2.98 | 6.02 | 6.34 | 118±4 | 3.69 | 100±16 | 16.5 |
|  | 2400 | 11.9 | 4.61 | 7.8 | 6.83 | 103±8 | 7.58 | 100±11.8 | 11.9 |
| EM | 0.25 | 9.74 | 1.83 | 8.97 | 1.08 | 94.1±9.2 | 9.74 | 106±6 | 5.68 |
|  | 2.5 | 8.94 | 1.54 | 7.13 | 5.18 | 95.5±6.4 | 6.72 | 111±3 | 2.86 |
|  | 200 | 6.93 | −1.34 | 7.27 | 2.4 | 121±6 | 4.65 | 108±17 | 16.2 |
|  | 640 | 7.25 | −1.14 | 6.83 | 0.9 | 117±8 | 7.13 | 90.6±25.4 | 28 |
| CH | 0.5 | 11.5 | −0.66 | 9.75 | 2.77 | 99.7±11.5 | 11.5 | 91.9±3.9 | 4.26 |
|  | 5 | 8.78 | 0.46 | 8.26 | 0.56 | 105±5 | 4.79 | 97.4±6.9 | 7.09 |
|  | 80 | 7.82 | −0.81 | 8.71 | −1.07 | 105±7 | 6.87 | 97.4±6.3 | 6.48 |
|  | 160 | 8.19 | 5.63 | 6.11 | 7.31 | 101±7 | 6.74 | 98.1±7.6 | 7.72 |
| PH | 0.5 | 10.7 | −0.62 | 8.29 | −0.53 | 102±11 | 10.7 | 93.7±5.0 | 5.34 |
|  | 5 | 8.41 | 1.45 | 8.08 | 0.12 | 109±8 | 7.69 | 92.1±6.4 | 6.89 |
|  | 80 | 9.23 | 1.68 | 8.43 | −2.45 | 97.6±8.6 | 8.82 | 95.5±11.1 | 11.7 |
|  | 160 | 6.35 | 6.44 | 5.62 | 7.47 | 101±6 | 6.35 | 100.1±11.1 | 11.1 |

**TABLE** **S4** Stability for seven key components in mice liver homogenates for PM hepatotoxicity study (*n* = 3).

| **Analyte** | **Concentration (ng/mL)** | **Freeze-thaw cycles** | | **Room-temperature (25ºC)** | | **Autosampler (15ºC)** | | **Long-term 20days**  **(−80ºC)** | |
| --- | --- | --- | --- | --- | --- | --- | --- | --- | --- |
|  |  | **RE (%)** | **RSD (%)** | **RE (%)** | **RSD (%)** | **RE (%)** | **RSD (%)** | **RE (%)** | **RSD (%)** |
| TSG | 1 | −0.2 | 6.82 | −2.73 | 3.97 | 3.05 | 8.15 | −6.51 | 4.9 |
|  | 3200 | 5.47 | 5.22 | 9.48 | 4.62 | 9.15 | 6.31 | 4.72 | 3.95 |
| EMG | 0.25 | 0.6 | 9.21 | −1.98 | 12.2 | 3.33 | 9.9 | 2.02 | 6.11 |
|  | 640 | −6.73 | 6.81 | −11.15 | 3.81 | −7.96 | 6.17 | −6.24 | 3.78 |
| AE | 0.5 | 0.52 | 8.95 | 7.19 | 11.8 | −4.66 | 8.49 | 3.65 | 3.25 |
|  | 160 | −2.07 | 8.82 | −5.68 | 9.17 | −7.64 | 9.54 | −2.67 | 10.6 |
| RE | 0.75 | −0.89 | 9.61 | −2.93 | 15.5 | −0.52 | 12.3 | 9.34 | 6.58 |
|  | 2400 | 5.34 | 7.32 | 3.72 | 5.72 | 9.98 | 3.43 | 5.12 | 7.62 |
| EM | 0.25 | 3.32 | 8.44 | 2.55 | 10.4 | −7.69 | 9.32 | 6.11 | 8.14 |
|  | 640 | −0.75 | 5.44 | −3.54 | 9.49 | 1.99 | 9.74 | 0.5 | 4.41 |
| CH | 0.5 | 3.96 | 5.17 | −2.17 | 14.3 | −0.79 | 4.7 | 7.92 | 2.40 |
|  | 160 | 7.97 | 4.14 | 6.04 | 6.6 | 8.76 | 3.28 | 5.17 | 4.37 |
| PH | 0.5 | 1.97 | 8.99 | −3.02 | 1.76 | −8.14 | 3.12 | 3.47 | 11.1 |
|  | 160 | 6.8 | 3.12 | 6.04 | 5.83 | 10.69 | 2.98 | 7.7 | 3.95 |

**TABLE S5** Serum and intrahepatic biochemical indexes in mice for PM hepatotoxicity study (Mean ± SD, *n* = 6).

| **Biochemical indexes** | | **CON** | | **PME** | **PMPE** | **PMPE-TSG** | **TSG** | **RME** | **RME-TSG** | **RMPE** | **RMPE-TSG** |
| --- | --- | --- | --- | --- | --- | --- | --- | --- | --- | --- | --- |
| *Serum* | ALT (U/L) | | 9.5±2.2 | 14.1±2.2 | 10.9±2 | 13.9±2.8 | 9.4±2.5 | 13.2±2.6 | 19.5±6.8 | 11.2±2.8 | 11.1±1.7 |
|  | TBA (μM) | | 5.6±1.1 | 8.8±2.6 | 7.9±2.6 | 8.4±1.8 | 8.4±2.1 | 6.7±1.8 | 8.8±2.5 | 5.8±1.6 | 7.6±2.8 |
|  | ALP (U/L) | | 50±10 | 46.1±10.9 | 33.1±3.7 | 38.7±3.5 | 36.8±4.9 | 25.9±4.4 | 30.2±7.1 | 25±5.5 | 23.9±6.2 |
|  | TG (mM) | | 1.5±0.5 | 1.4±0.3 | 1.6±0.4 | 1.8±0.7 | 1.5±0.4 | 1.3±0.3 | 1.2±0.4 | 1.2±0.4 | 1±0.3 |
|  | ALB (g/L) | | 20.4±1.7 | 20.8±1.7 | 21.3±1.8 | 19.2±3.1 | 21.6±4.2 | 20.7±3.7 | 18.7±2 | 20.1±2.9 | 17.8±0.5 |
|  | TP (g/L) | | 47.1±2.5 | 58.3±3.7 | 52.1±4.2 | 52.6±2.6 | 52.4±3.5 | 52.2±2.3 | 52.1±5.8 | 48.7±5.1 | 49.2±1.2 |
|  | GLB (g/L) | | 26.8±3.4 | 37.5±4.5 | 30.8±4.8 | 33.4±3.7 | 30.8±2 | 31.5±5.5 | 33.4±4.4 | 28.6±5.4 | 31.5±1.5 |
|  | CREA (μM) | | 26.3±9.6 | 45.7±11.1 | 21.8±3.3 | 24±7.1 | 21.9±3 | 36.2±21.3 | 30.7±18.7 | 17.4±4 | 22.3±2.9 |
| *Liver* | TNF-α (pg/mgprot) | | 58.1±16.7 | 131.8±44.3 | 93.2±19.5 | 124.1±47.4 | 110.5±48 | 73.7±15.1 | 78.2±13.4 | 52.8±14.4 | 63±7.6 |
|  | GSH (μmol/gprot) | | 8.8±1.5 | 6.6±0.4 | 7±0.6 | 6.1±1 | 10.1±2.1 | 10.8±3.1 | 12.7±3.6 | 11.4±2.3 | 13.6±3.1 |
|  | MDA (nmol/mgprot) | | 1.1±0.2 | 0.6±0.1 | 0.9±0.4 | 0.8±0.2 | 0.9±0.1 | 1.1±0.2 | 0.9±0.1 | 0.9±0.1 | 1±0.3 |
|  | GST (U/mgprot) | | 50.4±4.8 | 47.6±4.8 | 48.7±5.5 | 59.3±9.6 | 52.4±6.5 | 56.9±10.6 | 72.1±8 | 58.4±7 | 63.3±7.2 |

Each experimental group versus CON group, *: *p* < 0.05; **: *p* < 0.01; ***: *p* < 0.001.

**TABLE S6** Concentrations of emodin in mice tissues for PM hepatotoxicity study (Mean ± SD, *n* = 6).

| **Organ** | **Emodin (ng/g)** | | | | | | |
| --- | --- | --- | --- | --- | --- | --- | --- |
|  | **PME** | **PMPE** | **RME** | **RMPE** | **PMPE-TSG** | **RME-TSG** | **RMPE-TSG** |
| Brain | 37.7±21.9 | 1.89±0.28 | 5.62±5.51 | 7.13±4.44 | 27.6±8.4 | 1.96±2.14 | 3.25±2.57 |
| Heart | 173±106 | 49.5±30.8 | 15.1±13.7 | 12.2±12.3 | 8.47±2.98 | 39.7±35.2 | 1.26±1.73 |
| Spleen | 678±691 | 20.6±18.7 | 54.0±21.3 | 55.8±48.8 | 20.8±14.2 | 83.1±62.8 | 14.8±6.4 |
| Lung | 361±168 | 49.0±53.3 | 34.4±17.0 | 13.3±11.1 | 28.7±21.1 | 56.6±41.8 | 19.9±15.5 |
| Small Intstine | 5,628±3,848 | 519±400 | 438±452 | 113±22 | 413±289 | 636±575 | 491±284 |
| Large Intstine | 8,807±1,485 | 1,111±876 | 785±246 | 109±38 | 2377±2974 | 507±266 | 328±225 |
| Stomach | 15,817±9223 | 3,883±2,756 | 2,656±1,298 | 282±93 | 9,211±12,584 | 3,091±1,977 | 7,539±5,825 |
| Liver | 2,035±1,055 | 278±286 | 431±119 | 76.0±80.7 | 180±77 | 251±143 | 82.2±59.3 |
| Kidney | 1,253±505 | 98.1±111.5 | 91.8±53.1 | 18.4±5.6 | 48.3±28.3 | 151±83 | 76.4±90.1 |
| Serum | 182±111 | 7.06±3.47 | 7.37±1.76 | 2.35±1.36 | 13.4±6.5 | 8.51±6.87 | 3.27±2.33 |

**TABLE S7** Concentrations of TSG in mice tissues for PM hepatotoxicity study (Mean ± SD, *n* = 6).

| **Organ** | **TSG (ng/g)** | | | | | |
| --- | --- | --- | --- | --- | --- | --- |
|  | **PME** | **PMPE** | **TSG** | **PMPE-TSG** | **RME-TSG** | **RMPE-TSG** |
| Brain | 179±100 | 364±402 | 122±94 | 140±86 | ND | ND |
| Heart | 146±111 | ND | ND | ND | 48.1±30.2 | 0.23±0.22 |
| Spleen | 996±1,040 | 61.3±17.7 | 96.0±129.6 | 35.5±26.7 | 199±196 | 16.6±29.8 |
| Lung | 1,005±370 | 97.4±80.9 | 173±125 | 183±262 | 217±162 | 169±132 |
| Small Intstine | 27,750±19,271 | 367±351 | 3,756±6,650 | 1083±991 | 9,344±7,837 | 5,961±6,166 |
| Large Intstine | 18,659±17,886 | 176±90 | 996±1,184 | 180±205 | 509±493 | 223±216 |
| Stomach | 57,122±30,511 | 949±846 | 23,410±44,363 | 6,933±5,724 | 16,841±11,502 | 27,997±22,812 |
| Liver | 1,600±705 | 160±117 | 746±656 | 271±185 | 560±380 | 284±318 |
| Kidney | 1,140±700 | 52.9±69.8 | 181±302 | 111±99 | 261±157 | 568±875 |
| Serum | 544±515 | 43.6±11.4 | 221±72 | 80.1±20.2 | 96.0±80.0 | 46.7±29.7 |

**TABLE S8** Concentrations of emodin-8-*O-β*-D-glucoside in mice tissues for PM hepatotoxicity study (Mean ± SD, *n* = 6).

| **Organ** | **EMG (ng/g)** | | | | | | |
| --- | --- | --- | --- | --- | --- | --- | --- |
|  | **PME** | **PMPE** | **RME** | **RMPE** | **PMPE-TSG** | **RME-TSG** | **RMPE-TSG** |
| Brain | 9.43±10.46 | ND | ND | ND | ND | ND | ND |
| Heart | 9.17±15.36 | ND | ND | ND | ND | ND | ND |
| Spleen | 63.7±109.6 | ND | ND | ND | ND | ND | ND |
| Lung | 9.21±5.93 | ND | ND | ND | ND | ND | ND |
| Small Intstine | 480±566 | ND | 8.28±1.83 | ND | ND | 33.8±34.9 | ND |
| Large Intstine | 1,753±1,973 | ND | 43.6±223 | ND | ND | 140±118 | ND |
| Stomach | 8,016±3,434 | 327±486 | 491±360 | 49.9±32.0 | 362±585 | 933±648 | 226±238 |
| Liver | 39.3±28.7 | ND | 11.8±6.4 | ND | ND | 7.88±6.90 | ND |
| Kidney | 28.5±12.1 | ND | 3.20±4.20 | ND | ND | 3.34±2.15 | ND |
| Serum | 20.3±9.3 | ND | 1.48±0.79 | ND | ND | 1.36±0.89 | ND |

**TABLE S9** Concentrations of physcion in mice tissues for PM hepatotoxicity study (Mean ± SD, *n* = 6).

| **Organ** | **Physcion (ng/g)** | | | | | | |
| --- | --- | --- | --- | --- | --- | --- | --- |
|  | **PME** | **PMPE** | **RME** | **RMPE** | **PMPE-TSG** | **RME-TSG** | **RMPE-TSG** |
| Brain | 19.0±10.1 | 2.84±2.65 | 3.05±2.93 | 1.67±0.57 | 6.23±7.11 | 1.28±0.85 | 2.53±1.94 |
| Heart | 5.77±3.18 | 9.74±8.23 | 1.53±0.88 | 1.57±1.03 | 0.49±0.42 | 7.52±12.14 | 1.42±0.30 |
| Spleen | 66.4±87.4 | 3.18±2.65 | 4.13±1.95 | 20.8±22.8 | 3.09±2.40 | 17.2±22.0 | 2.92±1.39 |
| Lung | 25.1±13.8 | 18.7±38.6 | 3.57±2.23 | 2.44±2.54 | 5.23±6.93 | 14.8±15.7 | 8.66±9.03 |
| Small Intstine | 5,129±3,210 | 354±355 | 738±234 | 181±72 | 383±208 | 987±610 | 756±400 |
| Large Intstine | 4,811±1,838 | 857±654 | 485±282 | 136±82 | 1,362±1,133 | 397±120 | 600±477 |
| Stomach | 9,609±11,430 | 322±415 | 1,790±1,231 | 307±238 | 285.6±68.6 | 1,612±1,272 | 3,932±2,597 |
| Liver | 291±200 | 214±150 | 77.7±36.1 | 34.5±17.5 | 85.5±59.8 | 29.9±17.9 | 51.6±33.1 |
| Kidney | 180±112 | 36.0±27.1 | 9.18±4.45 | 4.05±2.60 | 16.0±7.8 | 22.9±13.7 | 29.9±48.0 |
| Serum | 9.29±3.68 | 0.98±0.79 | 0.43±0.23 | 0.33±0.04 | 1.08±0.69 | 2.78±2.26 | 0.42±0.29 |

**TABLE S10** Concentrations of aloe-emodin in mice tissues for PM hepatotoxicity study (Mean ± SD, *n* = 6).

| **Organ** | **Aloe-emodin (ng/g)** | | | | | | |
| --- | --- | --- | --- | --- | --- | --- | --- |
|  | **PME** | **PMPE** | **RME** | **RMPE** | **PMPE-TSG** | **RME-TSG** | **RMPE-TSG** |
| Brain | ND | ND | 5.49±3.13 | 5.21±2.13 | ND | 7.22±5.91 | 7.92±6.56 |
| Heart | ND | ND | 2.77±2.44 | 77.5±29.3 | ND | 18.9±23.1 | 67.9±59.3 |
| Spleen | ND | ND | 11.7±7.0 | 26.1±25.0 | ND | 31.0±22.9 | 3.25±2.37 |
| Lung | ND | ND | 9.70±6.30 | 8.26±0.0 | ND | 26.7±31.2 | 13.5±9.2 |
| Small Intstine | 69.4±103.8 | ND | 807±365 | 247±62.6 | 62.3±43.7 | 1,294±950 | 797±412 |
| Large Intstine | 20.1±8.6 | 50.5±54.0 | 507±188 | 241±71 | 105±70 | 539±235 | 327±278 |
| Stomach | 293±172 | 30.7±27.0 | 728±476 | 508±150 | 290±146 | 1,341±962 | 2,672±2,106 |
| Liver | ND | ND | 275±80 | 212±343 | ND | 216±141 | 116±59 |
| Kidney | ND | ND | 26.6±19.0 | 99.3±36.6 | ND | 88.7±38.6 | 89.4±152.2 |
| Serum | ND | ND | 18.4±15.6 | 77.0±31.3 | ND | 26.8±13.3 | 26.6±2.8 |

**TABLE S11** Concentrations of rhein in mice tissues for PM hepatotoxicity study (Mean ± SD, *n* = 6).

| **Organ** | **Rhein (ng/g)** | | | | | | |
| --- | --- | --- | --- | --- | --- | --- | --- |
|  | **PME** | **PMPE** | **RME** | **RMPE** | **PMPE-TSG** | **RME-TSG** | **RMPE-TSG** |
| Brain | ND | ND | ND | ND | ND | ND | ND |
| Heart | 23.0±9.8 | ND | 38.4±43.2 | ND | ND | 101±87 | ND |
| Spleen | 53.0±32.5 | ND | 38.2±32.0 | ND | ND | 89.0±80.5 | ND |
| Lung | 29.0±10.6 | ND | 50.9±28.1 | ND | ND | 161±126 | ND |
| Small Intstine | 118±91 | 11.3±8.2 | 1734±968 | 110±44 | 29.8±27.8 | 2,518±2,156 | 434±221 |
| Large Intstine | 178±99 | 21.3±17.2 | 842±195 | 171±114 | 12.3±2.7 | 783±539 | 216±153 |
| Stomach | 130±84 | 20.3±10.6 | 3,678±2,313 | 195±89.9 | 21.9±25.4 | 3,246±2,965 | 3,509±3,230 |
| Liver | 65.3±26.1 | ND | 955±365 | 311±313 | ND | 693±554 | 233±126 |
| Kidney | 25.9±9.6 | ND | 386±126 | 98.5±30.9 | ND | 780±554 | 304±284 |
| Serum | 10.1±5.2 | ND | 139±39 | 32.5±35.9 | ND | 522±981 | 60.6±43.4 |

**TABLE S12** Concentrations of chrysophanol in mice tissues for PM hepatotoxicity study (Mean ± SD, *n* = 6).

| **Organ** | **Chrysophanol (ng/g)** | | | | |
| --- | --- | --- | --- | --- | --- |
|  | **PME** | **RME** | **RMPE** | **RME-TSG** | **RMPE-TSG** |
| Brain | ND | 7.46±6.03 | 15.0±12.2 | 4.90±2.70 | 3.02±2.66 |
| Heart | ND | 14.7±5.8 | 13.8±8.0 | 50.1±55.6 | 5.23±5.55 |
| Spleen | ND | 29.4±6.6 | 21.9±12.3 | 100±99 | 64.9±84.9 |
| Lung | ND | 44.5±19.7 | 68.9±46.2 | 94±62 | 15.4±14.2 |
| Small Intstine | 44.2±55.1 | 3,305±1,153 | 3,678±1,915 | 3,611±2,082 | 379±118 |
| Large Intstine | 34.5±37.2 | 1,830±853 | 2,126±2,207 | 1,298±437 | 238±139 |
| Stomach | 178±266 | 5,541±2,996 | 8,407±6,625 | 5,019±4,415 | 518±232 |
| Liver | ND | 930±440 | 407±279 | 572±717 | 259±211 |
| Kidney | ND | 86.4±37.3 | 274±426 | 193±100 | 26.7±9.2 |
| Serum | ND | 10.5±3.8 | 13.7±13.9 | 25.6±15.8 | 5.48±1.10 |
